# Impact of various dopants (X = Zn, Mg, and Bi) on the structural, optical, and adsorption properties of (Co_0.8_Ni_0.1_X_0.1_)_3_O_4_ nanostructures

**DOI:** 10.1038/s41598-025-10965-4

**Published:** 2025-07-20

**Authors:** Ahmed Khalaf, Amani Aridi, Dema Dasuki, Marwa Elkady, Khulud Habanjar, Gehan M. El-Subruiti, Ramadan Awad

**Affiliations:** 1 Physics Department, Faculty of Science, Damanhour University, Damanhour, Egypt; 2 Public Health Department, Faculty of Health Sciences, Modern University for Business and Science, Beirut, Lebanon; 3 Physics Department, Faculty of Science, Beirut Arab University, Beirut, Lebanon; 4 Fabrication Technology Research Department, Advanced Technology and New Materials Research Institute (ATNMRI), City of Scientific Research and Technological Applications (SRTA-City), New Borg El-Arab City, 21934 Alexandria Egypt; 5 Chemical and Petrochemical Engineering Department, Egypt-Japan University of Science and Technology (E-JUST), New Borg El-Arab City, Alexandria Egypt; 6 Chemistry Department, Faculty of Science, Alexandria University, Alexandria, Egypt; 7 Physics Department, Faculty of Science, Alexandria University, Alexandria, Egypt; 8 Department of Basic Sciences, Faculty of Computer Science and Artificial Intelligence, Pharos University in Alexandria, Alexandria, Egypt

**Keywords:** Tricobalt tetraoxide (Co_3_O_4_), Co-precipitation method, Adsorption, Methylene blue, Wastewater treatment, Chemistry, Materials science, Physics

## Abstract

This study aimed to enhance the adsorption efficiency of spinel Co_3_O_4_ against methylene blue dye removal, a significant environmental treatment. Hence, metal elements (X = Zn, Mg, and Bi) doped (Co_0.8_Ni_0.1_ X _0.1_)_3_O_4_ nanoparticles (NPs) were synthesized by the co-precipitation method, with a crystallite size range between 17 and 23 nm. The X-ray diffraction (XRD) analysis with the Rietveld refinement confirmed the spinel single-phase for Zn and Mg-doped (Co_0.9_Ni_0.1_)_3_O_4_ NPs without any secondary phases. However, the Bi-doped (Co_0.9_Ni_0.1_)_3_O_4_ NPs exhibited a secondary BiOCl phase, indicating the lack of Bi ions incorporation into the (Co_0.9_Ni_0.1_)_3_O_4_ lattice. Accordingly, the Fourier transform infrared spectroscopy (FTIR) confirmed the BiOCl secondary phase, and X-ray photoelectron spectroscopy (XPS) analysis verified the formation of the spinel structure in all samples. Morphologically, the scanning electron microscope (SEM), energy-dispersive X-ray (EDX), and transmission electron microscope (TEM) of doped samples revealed the presence of agglomerated particles with spherical and hexagonal nanoparticles. Subsequent investigations with high-resolution resolution-(HRTEM) and selected-area electron diffraction (SAED) demonstrated that high crystalline spinel structures. The Raman spectra exhibited vibrational modes related to the (Co_0.9_Ni_0.1_)_3_O_4_ cubic structure. The optical band gap increased with Mg-doping, and decreased with Bi-doping as compared to the Zn-doped sample. The PL intensity of Zn-doped (Co_0.9_Ni_0.1_)_3_O_4_ was lower than Mg and Bi samples, indicating the slower recombination rate of photogenerated charge carriers in the Zn-doped sample. Eventually, the highest adsorption capacity of 94.4 mg.g^−1^, was reached by the Zn-doped (Co_0.9_Ni_0.1_)_3_O_4_ NPs. Afterward, the adsorption behavior was studied by changing the contact time, initial dye concentration, and pH. The adsorption of methylene blue onto the synthesized adsorbents was best described by the Freundlich isotherm model. These findings highlight the promising performance of the prepared NPs, supporting their potential application as effective adsorbents for water treatment.

## Introduction

Spinel oxides are recognized as one of the major classes of ceramic oxides. Among them, tricobalt tetroxide (Co₃O₄) stands out as a typical normal spinel. It is characterized by cobalt ions in two oxidation states: Co²⁺ occupying the tetrahedral CoO₄ sites, and Co³⁺ located in the octahedral CoO₆ sites^1^. Moreover, Co_3_O_4_ is a very important p-type semiconductor magnetic material with various morphological structures, such as nanorods, nanosheets, and ordered nano flowers that have been synthesized^2–5^. Many researchers have employed a range of synthetic methods and nanostructuring techniques to achieve superior performance in applications such as catalysis, supercapacitors, battery electrodes, sensors, drug delivery, and the electrical industry^6^. An effective approach involves doping or including impurities in spinel; Co_3_O_4_ is one of the most effective strategies. In this regard, substituting Co atoms in spinel Co_3_O_4_ with a relatively less expensive and eco-friendly metal element like Ni, Cu, Mn, Zn, or Mg can significantly affect the microstructure, nanostructure, and electronic properties of the spinel. Specifically, nickel is a typical transition metal element and an attractive dopant due to its cost-effectiveness, less toxicity, and favorable redox properties (Ni^2+^/Ni^3+^)^7^. Moreover, the ionic radius of nickel is nearly identical to that of cobalt. This means that even small amounts of Ni substitution can enhance crystallinity, modify the energy gap, and improve the electrical properties of cobalt. The incorporation of Ni into the cobalt structure can also facilitate the formation of oxygen vacancies, which positively impacts photo-sensing performance. Ren et al.^8^ demonstrated that Ni doping within the spinel lattice of Co_3_O_4_ enhances the reaction kinetics and allows low-temperature oxidation by promoting the activity of lattice oxygen and creating oxygen defects on the catalyst surface. Furthermore, Barbouch et al.^9^ reported that Zn doping in Co_3_O_4_ thin films resulted in a significant enhancement of optical absorption and electrical conductivity, suggesting the creation of voids and/or the replacement of Co²⁺ ions by Zn²⁺ ions within the film structure. Due to their thermochemical stability, good conductivity, and low production cost, Mg²⁺ ions are also commonly doped into Co_3_O_4_ NPs^10^. Sundararajan et al.^10^ studied the effect of Mg^2+^ ions doping on the structural and physical properties of Co_3_O_4_ NPs. Their findings indicated that Co_3_O_4_ and Mg_0.1_Co_0.9_O_4_ exhibit a single-phase, cubic structure with the space group *Fd*3*m*. On increasing the Mg^2+^ content, a two-phase system with cubic (Co_3_O_4_) and hexagonal (MgO) was formed due to the substitution of Co^2+^ by Mg^2+^ ions, resulting in the composite formation. However, co-doping introduces two or more dopants simultaneously, which can lead to synergistic effects that significantly enhance the material’s properties. Deeloed et al.^11^ investigated the impact of Zn and Ni co-doping on the structure and magnetization of Zn_1−x_Ni_x_Co_2_O_4_ spinels synthesized via a hydrothermal approach. They found that samples with higher zinc content (x = 0.00 and 0.25) exhibited crystallinity levels comparable to pure Co₃O₄, likely due to the structural similarity between ZnCo_2_O_4_ and CoCo_2_O₄, both having a normal-type spinel structure. In contrast, samples with higher nickel substitutions showed a noticeably lower degree of crystallinity. Besides, increased Ni content correlated with enhanced magnetization values.

On the other hand, water pollution has become one of the most pressing environmental crises today, largely stemming from the uncontrolled disposal of pharmaceutical and industrial waste^12,13^. The presence of various pollutants in water poses serious threats to both human health and aquatic ecosystems^14–16^. Recently, a rise in the textile pollutant count was detected, such as the cationic dyes (methylene blue, malachite green, rhodamine B, etc.) and azo dyes (Congo red, methyl orange, reactive blue 19, etc.)^16–18^. Methylene blue, an aniline-based cationic dye with the chemical formula C_16_H_18_ClN_3_S, is widely used in industry as a dark blue dye for cotton and silk^19^. As previously reported, MB dye can cause several adverse health effects in humans, including cyanosis, vomiting, shock, and an increased heart rate^20^. In the environment, methylene blue negatively impacts plant life, leading to growth inhibition and a reduction in pigment and protein content in microalgae^20^. These harmful effects highlight the urgent need for effective removal of methylene blue from wastewater before it is discharged into the environment. To tackle this, nanoparticles have been employed as adsorbents in various wastewater treatment techniques, particularly in physical methods such as adsorption^21^. Among these, Co_3_O_4_ nanoparticles have gained attention for their excellent dye adsorption performance, owing to their unique physical properties^22^. It is noteworthy that the Co_3_O_4_ nanoparticles reported an outstanding dye adsorbing performance, under specific conditions^22^. Co_3_O_4_ nanoparticles exhibited an adsorption capacity of 46.08 mg/g for methylene orange dye at room temperature^23^. Moreover, a significant enhancement of the adsorption performance was marked upon the doping of cobalt in Co_3_O_4_^24^.

Furthermore, as mentioned previously, co-doping Co_3_O_4_ with Cu-Ni and Cd-Ni significantly enhances its ability to degrade methyl orange dye, compared to undoped or singly Ni-doped Co_3_O_4_^25^. This improvement is largely attributed to the synergistic effects introduced by the co-dopants, which can modify the electronic structure, increase surface area, and enhance active sites for dye interaction. Notably, Cd-Ni co-doped Co_3_O_4_ exhibited the most remarkable performance, achieving an impressive 93% degradation efficiency of MO dye. These results highlight the potential of co-doping strategies to optimize the catalytic properties of Co_3_O_4_ nanoparticles for effective wastewater treatment.

Herein, the novelty lies in the significant effect of co-doping metal elements in oxide NPs to enhance the MB dye adsorption capacity. For this purpose, a simple, eco-friendly, and low-cost synthesis technique was utilized. As for reliability, the synthesized nanoparticles were tested repeatedly through the adsorption cycle, confirming their stability and reusability. Additionally, the synthesized nanoparticles performed the highest adsorption capacity compared to previous literature.

As mentioned, the main advantages of this study are the eco-friendly synthesis technique along with the significant wastewater treatment results. However, a real water sample shall be evaluated to scale up the challenges.

So, a co-doping strategy was chosen where the concentration of one of the dopants is kept constant and the other varies. As for this, selected metal elements (X = Zn, Mg, and Bi) doped (Co_0.8_Ni_0.1_ X _0.1_)_3_O_4_ NPs were developed via the co-precipitation method. The effects of Zn, Mg, and Bi as dopants on the structural, morphological, and optical properties of (Co_0.9_Ni_0.1_)_3_O_4_ were investigated. Different techniques were used, such as XRD, FTIR, XPS, SEM, EDX, TEM, HRTEM, SAED, Raman, UV-Vis, and PL spectroscopy. Furthermore, evaluating the adsorption performance of Zn, Mg, and Bi-doped (Co_0.9_Ni_0.1_)_3_O_4_ nanoparticles in dark conditions has proven its effectiveness against a known pollutant, methylene blue.

## Experimental details

### Materials

For the fabrication of (Co_0.8_Ni_0.1_X_0.1_)_3_O_4_ nanoparticles, high-purity precursor chlorides were purchased and used without additional purification. The following starting materials were purchased from Sigma-Aldrich (USA): nickel (II) chloride hexahydrate (NiCl_2_.6H_2_O, ≥ 98%), zinc chloride (ZnCl_2_, 98-100.5%), magnesium chloride hexahydrate (MgCl_2_.6H_2_O, ≥ 99%), and bismuth (III) chloride (BiCl_3_, ≥ 98%). However, cobalt (II) chloride hexahydrate (CoCl_2_.6H_2_O, 98-99.9%) was purchased from Acros Organics (Belgium).

### Synthesis route

**Fig. 1:**
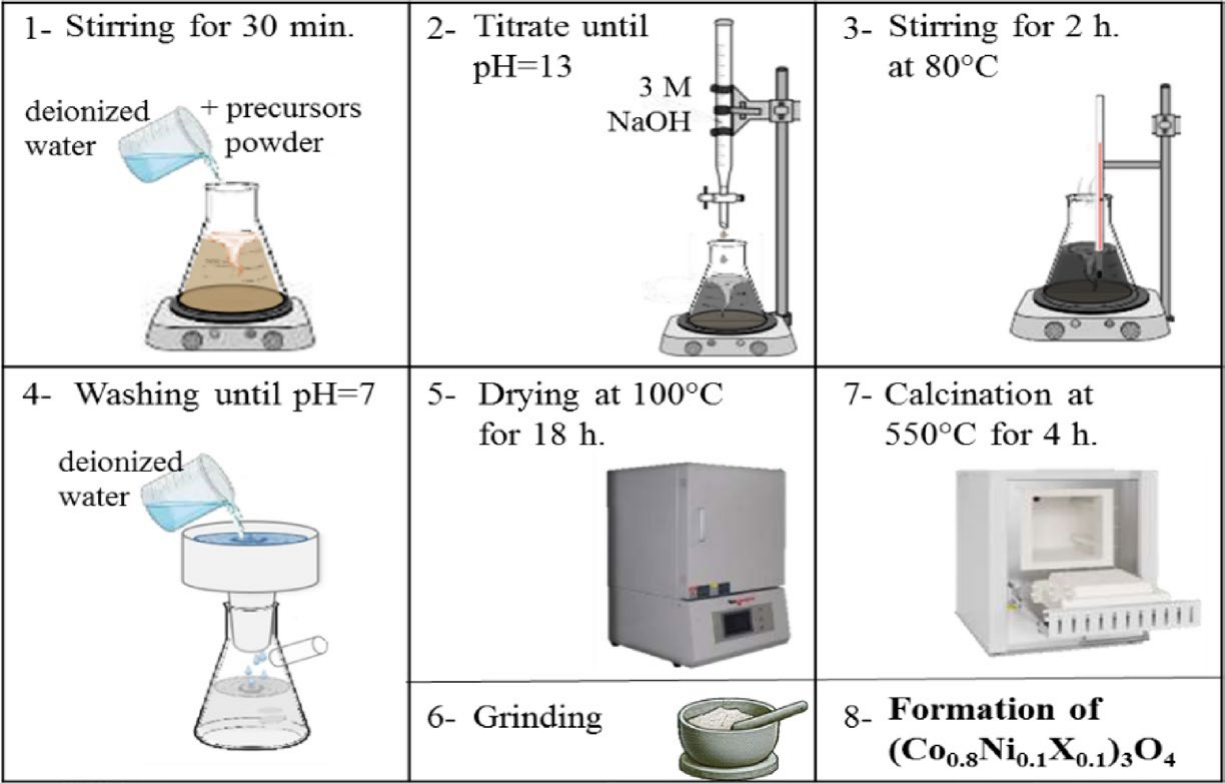
Schematic diagram illustrates the co-precipitation synthesis technique of (Co_0.8_Ni_0.1_ X _0.1_)_3_O_4_ nanoparticles.

Figure 1 shows the diagram for the typical co-precipitation method for the synthesis of metal (X = Zn, Mg, and Bi) doped (Co_0.8_Ni_0.1_X_0.1_)_3_O_4_ nanoparticles^26^. First, the precursor chlorides were mixed and stirred for 30 min to form a one molar solution. Following this, the pH value of the solution increased to 12 by dropping over three molar sodium hydroxides. Then, the solution was heated for 2 h at 353 K while maintaining constant stirring. The formed precipitate was then washed with deionized water until a neutral medium (pH = 7) was reached. The collected precipitate was dried for 18 h at 373 K and finally calcined for 4 h at 823 K to produce (Co_0.8_Ni_0.1_Mg_0.1_)_3_O_4_ nanoparticles. The same method was used to synthesize (Co_0.8_Ni_0.1_Mg_0.1_)_3_O_4_ and (Co_0.8_Ni_0.1_Bi_0.1_)_3_O_4_ nanoparticles, except that MgCl_2_·6 H_2_O and BiCl_3_ were used as precursors under the same conditions.

### Characterization studies

The X-ray diffraction (XRD) analysis and phase purity were performed using a Bruker D8 diffractometer through $\:Cu\:K\alpha\:$ radiation ($\:\lambda\:=0.154\:nm$). Afterward, the Thermo Scientific Nicolet iS5 FTIR spectrometer was utilized to obtain the FTIR studies, operating between $\:500-4000\:c{m}^{-1}$. The elemental composition, X-ray photoelectron spectroscopy, was examined by K-Alpha (Thermo Fisher Scientific, USA), using monochromatic X-ray Al Kα radiation with $\:h\nu\:=1486.6\:eV$. However, the Energy-dispersive X-ray (EDX) was assessed by a X-ray microanalyzer attached to a JEOL JSM-5500LV at 20 kV for the Scanning electron microscope studies, operating at 20 kV. Transmission electron microscope (TEM) images were captured using JEOL JEM-1400 plus operated at 80 kV, while the High-resolution transmission electron microscope (HR-TEM) images were recorded using JEM-2100 Plus electron microscope. Additionally, Raman studies were obtained by a confocal microscope using a laser excitation source (532 nm). The textural properties were determined using N_2_ adsorption–desorption isotherms, utilizing a Micromeritics 3Flex Surface Area Analyzer (Micromeritics Instrument Corp.). The optical studies, UV-vis and Photoluminescence spectroscopy were obtained by JASCO FP-8500 spectrophotometer and JASCO FP-8600 spectrofluorometer ($\:{\lambda\:}_{ex}=325\:nm)$, respectively.

### Adsorption experiment

The adsorption capacity of the synthesized materials was assessed for removing methylene blue dye under dark conditions. The experiments were conducted by mixing 50 mg of synthesized samples with 100 mL of a 50 mg/L methylene blue solution. Subsequently, the effect of initial dye concentration on adsorption was examined within the range of 5 to 100 mg/L. To evaluate the effect of pH, the dye solution was adjusted within a pH range of 1 to 11 using 0.1 N hydrochloric acid (HCl) for acidic conditions and 0.1 N sodium hydroxide (NaOH) for alkaline conditions. The adsorption capacity was quantified through photometric analysis using a Shimadzu UV mini 1240 V spectrophotometer, with the maximum absorption wavelength for methylene blue recorded at 664 nm. The percentage of dye removal was calculated using the following equation^27^:

1 $$\:Removal\:\:\%\:=\:\frac{{C}_{i}\:-\:{C}_{f}}{{C}_{i}}\:\times\:100\:$$

where *C*_i_​ and *C*_f_​ represent the initial and final methylene blue concentrations, respectively. The adsorption capacities of the adsorbents were calculated at equilibrium and at specific time intervals, expressed by the equations^27^:

2 $$\:{q}_{\text{e}}=\:\frac{{C}_{\text{i}}\:-\:{C}_{\text{e}}}{m}\:\times\:V\:\:and$$

3 $$\:{q}_{\text{t}}=\:\frac{{C}_{i}\:-\:{C}_{t}}{m}\:\times\:V.$$

where *C*_e_​ and *C*_t_​ denote the equilibrium and time-dependent dye concentrations, respectively. Furthermore, V represents the solution volume, while m corresponds to the mass of the adsorbent.

## Results and discussion

### XRD analysis

**Fig. 2:**
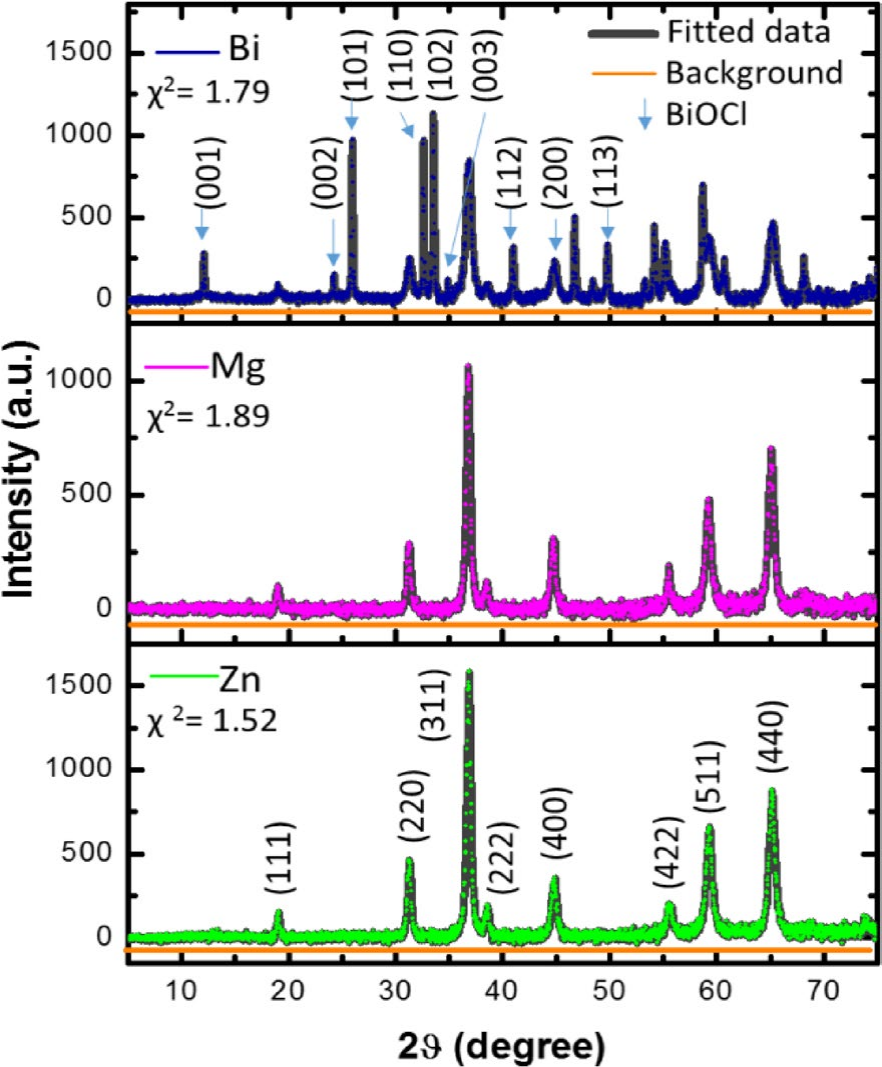
Rietveld refinement fitted XRD patterns for Zn, Mg, and Bi-doped (Co_0.8_Ni_0.1_ X _0.1_)_3_O_4_ nanoparticles.

**Fig. 3:**
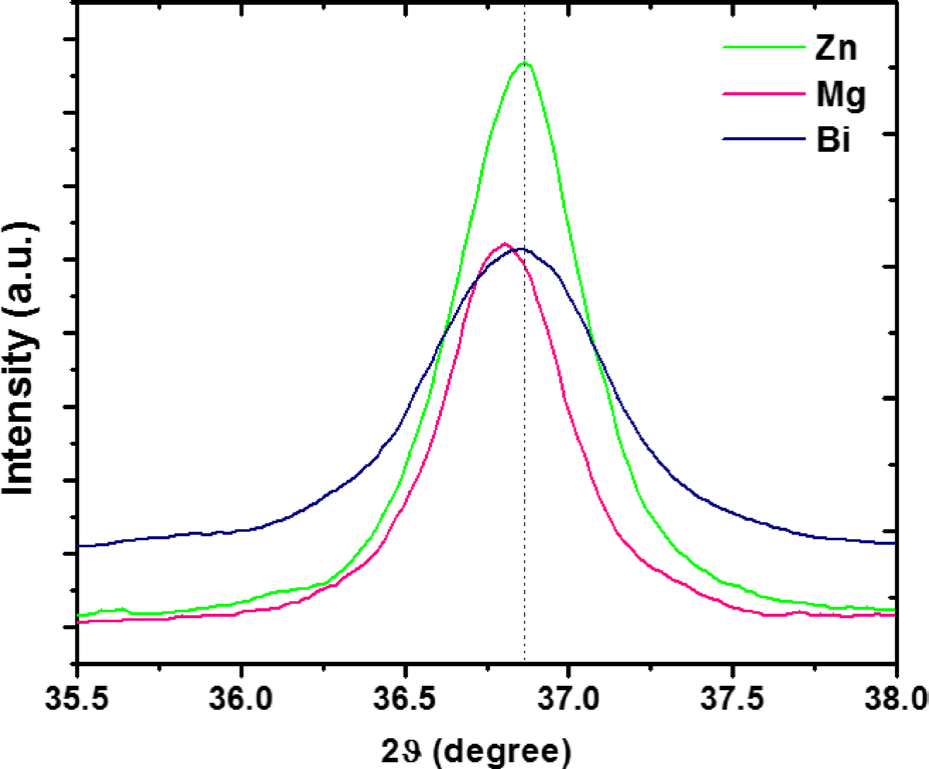
Evaluation of the effect of dopant elements on the intensity of the (311) diffraction peak.

Figure 2 depicts the XRD patterns with Rietveld refinement of (Co_0.8_Ni_0.1_ X _0.1_)_3_O_4_, with X = Zn, Mg, and Bi nanoparticles. Phase identification was performed using the Materials Analysis Using Diffraction (MAUD) software (version 2.999) developed by Lutterotti^28^. XRD patterns of all metal-doped samples revealed characteristic diffraction peaks corresponding to the Co_3_O_4_ cubic spinel structure with a lattice constant of 8.084 Å (space group $\:Fd3m$, JCPDS: NO. 043:1003)^1^. The observed peaks correspond to the reflection at (111), (220), (311), (400), (511), and (440) lattice planes. However, the (Co_0.8_Ni_0.1_Zn_0.1_)_3_O_4_ and (Co_0.8_Ni_0.1_Mg_0.1_)_3_O_4_ samples have a single cubic phase$\:,$ without any secondary phases, involving metals or metal oxides, attesting to the high purity of the obtained phases. On the other hand, the Bi-doped (Co_0.9_Ni_0.1_)_3_O_4_ sample exhibited two distinct phases. The primary phase was the cubic spinel structure of (Co_0.9_Ni_0.1_)_3_O_4,_ accompanied by multiple additional peaks corresponding to bismuth oxychloride (BiOCl) as a secondary phase. The BiOCl phase possesses a tetragonal structure (space group *P4/nmm*, JCPDS NO. 06-0249)^29^. The formation of the BiOCl phase might be due to the hydrolysis of BiCl_3_ during the growth process^30^. It is worth mentioning that BiOCl is a unique semiconductor photocatalyst with a two-dimensional (2D) layered structure, composed of two layers of Cl ions intermixed with one layer of [Bi_2_O_2_]^29^. Similar findings were reported for Bi-doped TiO_2_ nanoparticles synthesized using the hydrothermal method^31^. Besides, the high-intensity diffraction peaks also indicate the outstanding crystallinity of the fabricated (Co_0.9_Ni_0.1_)_3_O_4_ nanoparticles. Specifically, the high-intensity diffraction peak at 2θ = 36.6˚ shows the preferred growth of the (311) crystal plane of the cubic phase (Co_0.9_Ni_0.1_)_3_O_4_^32^. Figure 3 shows that for the Zn-doped sample, the intensity of the (311) diffraction peak increases, while the full width at half maximum (FWHM) decreases. This suggests an enhancement in crystallinity, reflected by larger crystallite size and reduced internal microstrain compared to the Mg and Bi-doped samples.

The quality of the Rietveld refinement is assessed using reliability parameters, including the goodness of fit (χ²), weighted profile (R_wp_), Bragg R-factor (R_b_), and expected R-value (R_exp_), which are provided in Table 1. These parameters show a good agreement between the simulated and experimental patterns. Additionally, the phase fractions and lattice parameters obtained from the Rietveld refinement are also listed in Table 1. The phase fractions for (Co_0.8_Ni_0.1_Zn_0.1_)_3_O_4_ and (Co_0.8_Ni_0.1_Mg_0.1_)_3_O_4_ nanoparticles indicate the formation of the single-phase spinel structure, confirming that Zn and Mg are successfully incorporated into the (Co_0.8_Ni_0.1_ X_0.1_)_3_O_4_ crystal structure. In contrast, Bi-doped (Co_0.8_Ni_0.1_ X _0.1_)_3_O_4_ displayed a two-phase composite system, consisting of 88.65% of (Co_0.9_Ni_0.1_)_3_O_4_ and 11.35% of BiOCl. Moreover, the lattice parameters for Zn-doped and Mg (Co_0.8_Ni_0.1_ X _0.1_)_3_O_4_ are higher than those of both Bi-doped (Co_0.8_Ni_0.1_X_0.1_)_3_O_4_ nanoparticles.

The variation in the lattice parameter can be explained by considering the ionic radii difference of Zn^2+^ ions (0.74 Å) and Mg^2+^ ions (0.72 Å) against Co^2+^ ions (0.745 Å) and Co^3+^ ions (0.68 Å) in tetrahedral and octahedral coordination sites, respectively^33^. Although the lattice parameter was expected to decrease with Mg²⁺ substitution according to Vegard’s law, the observed increase may be due to variations in the Mg²⁺ substitution rates at tetrahedral and octahedral sites. In the case of Bi-doped (Co_0.8_Ni_0.1_X_0.1_)_3_O_4_ nanoparticles (Bi^3+^= 0.96 Å)^34^, the decrease in the lattice parameter is likely due to the Bi^3+^ ions that do not integrate into the spinel lattice, instead forming BiOCl on the (Co_0.9_Ni_0.1_)_3_O_4_ surface, leading to a reduction in the lattice parameter.

The crystallite sizes of Zn, Mg, and Bi-doped (Co_0.8_Ni_0.1_X_0.1_)_3_O_4_ were estimated using Scherrer’s formula using the major peak of the (311) plane^35^:

4 $$\:D=\frac{0.9\lambda\:}{\beta\:\:cos\theta\:},$$

where, $\:2\theta\:$ is the Bragg’s diffraction angle, and is the FWHM. Using the crystallite sizes, the dislocation density $\:\delta\:\:$(the length of dislocation lines/unit volume) can be evaluated using the formula^36^:

5 $$\:\delta\:=\frac{1}{{D}^{2}}$$

The values of the crystallite sizes and dislocation density are also included in Table 1. The crystallinity and the crystallite size *were* influenced by factors such as doping, temperature, and the synthesis method of the Co_3_O_4_. Sarfraz et al.^37^, reported that Co_3_O_4_ treated at 550 °C showed a crystallite size of 27 nm. However, Abdallah et al.^38^, found that a higher temperature of 700 °C resulted in much larger Co_3_O_4_ crystallites, about 31 nm. Nevertheless, doping (Co_0.8_Ni_0.1_ X _0.1_)_3_O_4_ nanoparticles with Zn²⁺ or Mg²⁺ promotes crystal growth, as these ions are similar in size to Co²⁺ and Ni²⁺, allowing them to integrate easily into the lattice without causing much distortion. In contrast, doping with Bi³⁺, which has a larger ionic radius and higher charge, leads to the formation of a BiOCl secondary phase on the surface of (Co_0.9_Ni_0.1_)_3_O_4_ nanoparticles. This secondary phase inhibits crystal growth, reduces the crystallite size, and introduces many structural defects, resulting in an increased dislocation density.

**Table 1:** The refinement parameters, phase fraction, lattice parameters, crystallite size, and dislocation density.

Sample	$\chi ^{2}$	*R*_wp_%	*R*_b_%	*R*_exp_%	Phase%		a (Å)	D (nm)	δ × 10^15^ (line/m^2^)
					Spinel Phase	BiOCl			
(Co_0.8_Ni_0.1_Zn_0.1_)_3_O_4_	1.52	18.60	14.53	11.76	100%	--	8.194	16.08	4.63
(Co_0.8_Ni_0.1_Mg_0.1_)_3_O_4_	1.79	24.63	19.17	13.72	100%	--	8.108	16.67	5.93
(Co_0.8_Ni_0.1_Bi_0.1_)_3_O_4_	1.89	21.64	16.81	11.41	88.65%	11.35%	8.097	11.77	4.39

### FTIR spectroscopy

**Fig. 4:**
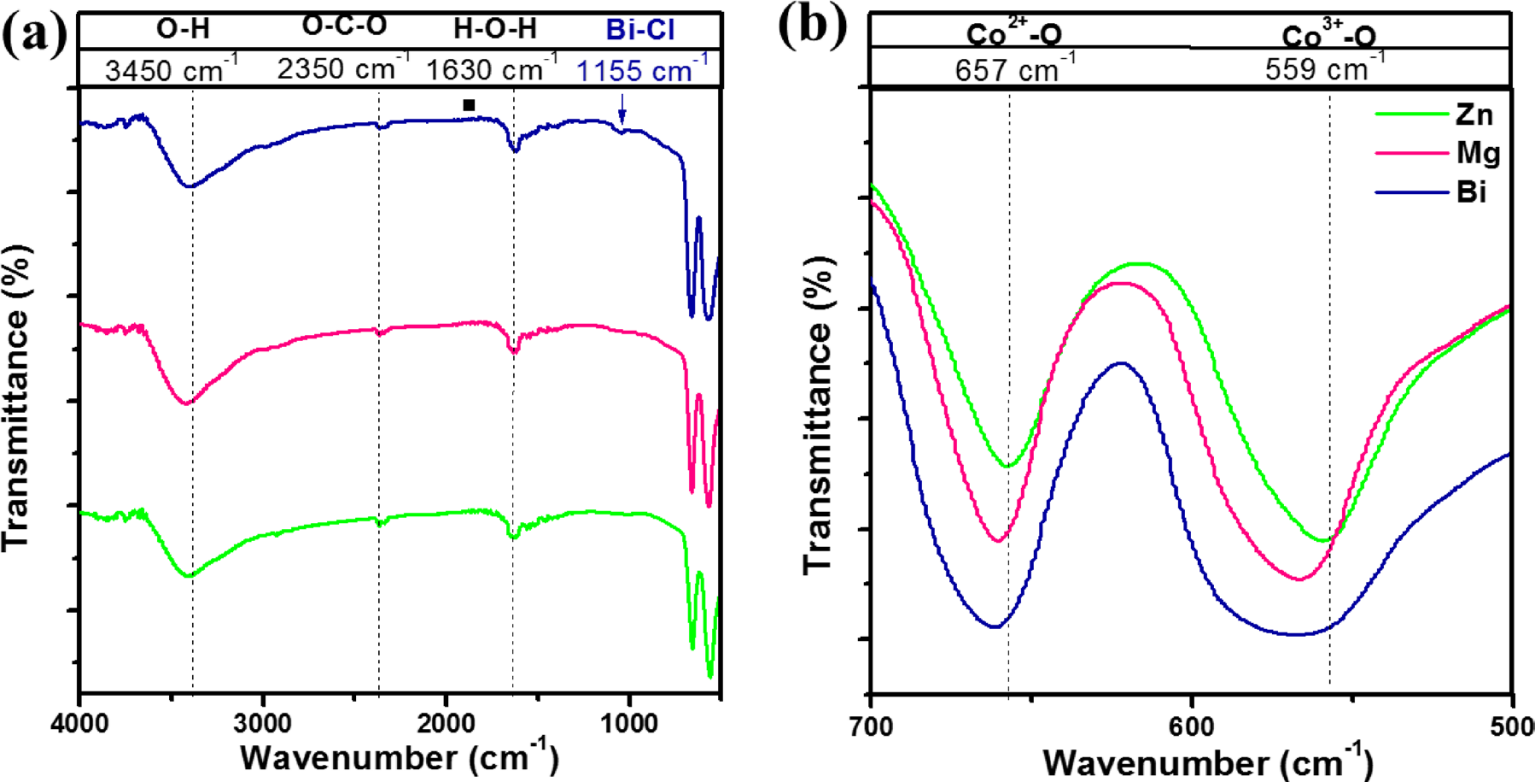
(a) The full FTIR spectra for Zn, Mg, and Bi-doped (Co_0.8_Ni_0.1_ X _0.1_)_3_O_4_ nanoparticles spectral region from 400 to 4000 cm^−1^, and (b) the magnified image of the 500–700 cm^−1^ spectral region.

To confirm the functional groups, the FTIR analysis of Zn, Mg, and Bi-doped (Co_0.8_Ni_0.1_ X _0.1_)_3_O_4_ nanoparticles was performed. As shown in Fig. 4, the FTIR spectra were carried out in the absorption range of 4000–500 cm^−1^. As the synthesized samples collect moisture from the air, the two absorption bands near 3450 and 1630 cm^−1^ are indicative of the stretching and bending vibrations of water molecules (O-H), respectively^39^. The peak at about 2370 cm^−1^ characterizes the asymmetric vibration (C = O) of CO_2,_ which was absorbed from the air. The two strong characteristic bands around 574 cm^−1^ and 666 cm^−1^, observed in all samples, can be ascribed to the stretching vibration of M-O (M = Co, Ni, Zn, Mg, or Bi) bonds within the Co_3_O_4_ spinel structure. The peak around 574 cm^−1^ corresponds to OB_3_ vibration in the spinel lattice [B donates Co^3+^/Ni^3+^ ion occupying the octahedral site]. Likewise, the second band 666 cm^−1^ corresponds to ABO_3_ vibration [A represents Co^2+^ ions in the tetrahedral site]^40^. In Mg and Bi-doped samples, the M-O band slightly shifts to higher wavenumbers compared to the Zn-doped sample. This shift may be associated with the decrease in the lattice parameters of Mg and Bi-doped samples. The peak around 1155 cm^−1^ can be attributed to the Bi-Cl bond for the formation of the BiOCl phase with the Bi-doped sample. These observations confirmed the formation of a single phase of Zn and Mg-doped (Co_0.8_Ni_0.1_X_0.1_)_3_O_4_ with a cubic spinel structure. This is in good agreement with XRD analysis.

### XPS spectroscopy

**Fig. 5:**
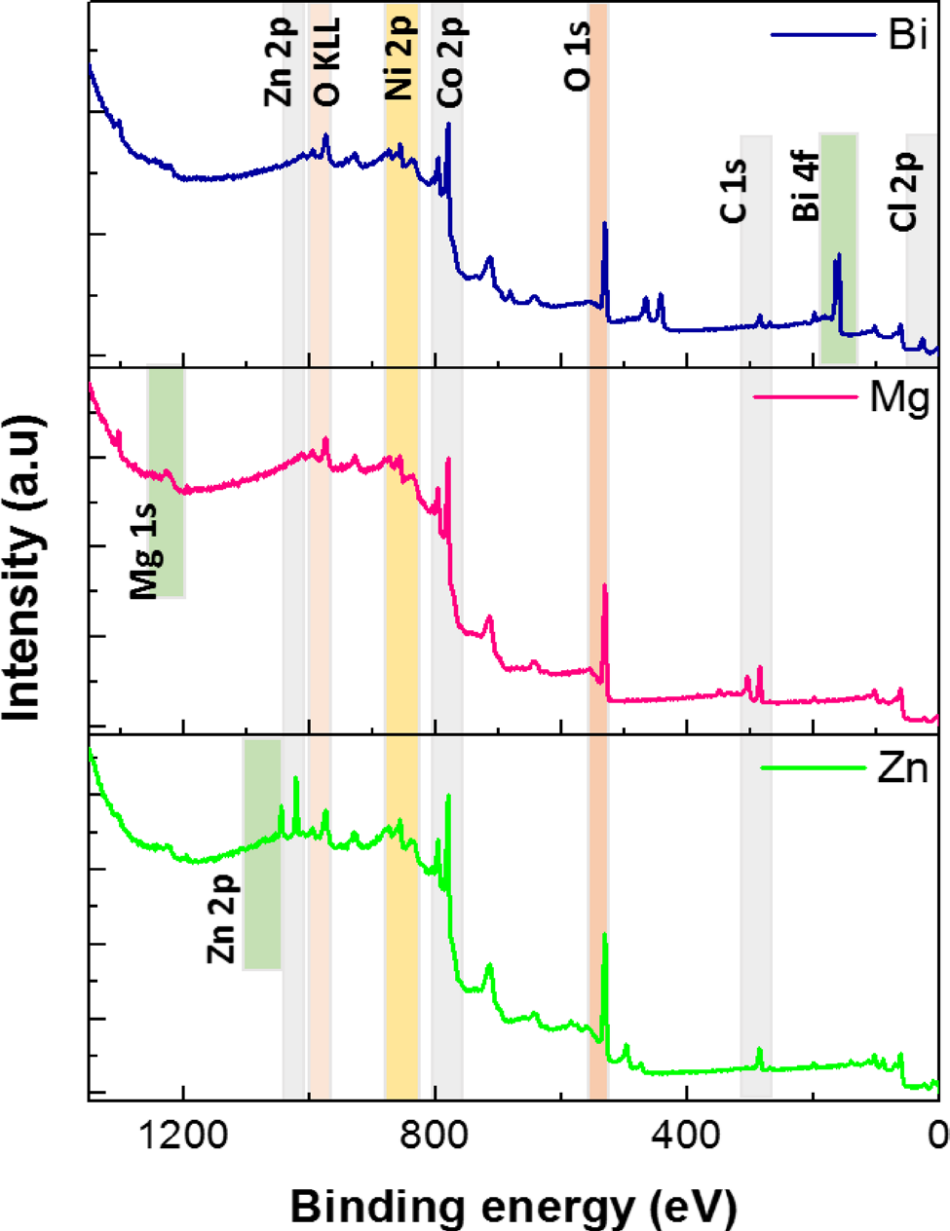
The XPS full survey spectra for (Co_0.8_Ni_0.1_X_0.1_)_3_O_4_ nanoparticles.

The XPS measurements were carried out to study the composition and investigate the electronic structure of Zn, Mg, and Bi-doped (Co_0.8_Ni_0.1_ X _0.1_)_3_O_4_ samples. Figure 5 (a) presents the full survey spectra for three investigated samples in the binding energy range 0 to 1350 eV. The binding energies of the particles were calibrated by taking the C1s peak as reference (285 eV). The XPS spectra show all the expected peaks, and no additional elements were detected other than the appearance of Cl in the Bi-doped sample. The photoemission and Auger peaks are labelled accordingly in the figure.

**Fig. 6:**
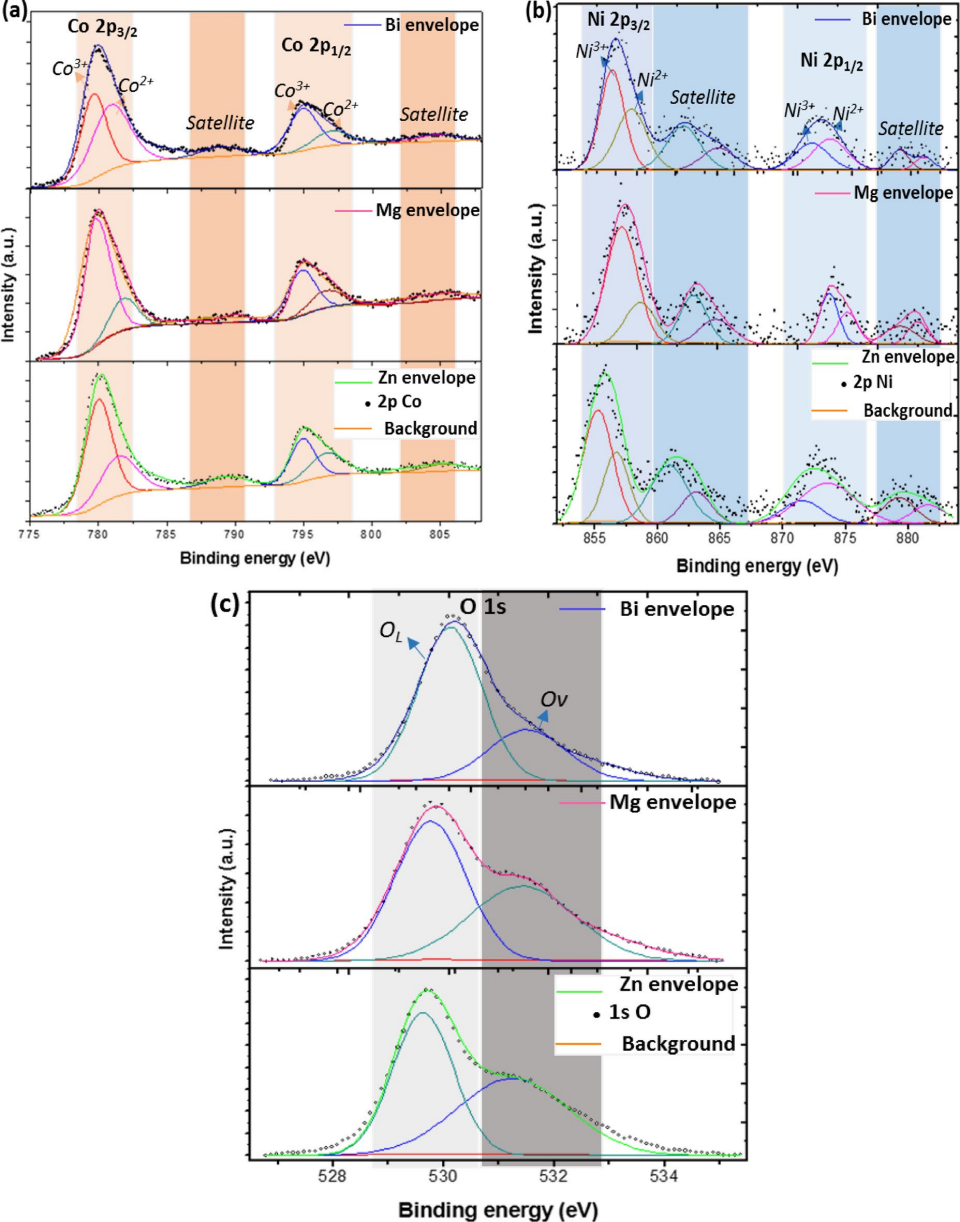
High-resolution XPS spectra of (a) Co, (b) Ni, and (c) O, for (Co_0.8_Ni_0.1_X_0.1_)_3_O_4_ nanoparticles.

Figure 6a presents the high-resolution Co 2p XPS spectra for Zn, Mg, and Bi-doped samples. The Co 2p region is composed of two main peaks corresponding to the spin-orbit components Co 2p_3/2_ and Co 2p_1/2_. Additionally, two satellite peaks appear at higher binding energies relative to the main peaks. The fitted peaks at 781.4 eV and 796.8 eV are attributed to the Co^2+^ species, while those at 780.7 eV and 794.8 eV correspond to Co^3+^. The fitted peaks at 781.4 eV and 796.8 eV are attributed to the Co^2+^ species, while those at 780.7 eV and 794.8 eV correspond to Co^3+^. Moreover, shake-up satellite peaks are observed at 788.97 eV and 803.99 eV. These results indicate the coexistence of both Co^2+^and Co^3^⁺ oxidation states in all metal-doped samples^41^. Besides, the peaks at 788.97 eV and 803.99 eV were attributed to the shake-up satellite peaks. This suggests that both the Co^2+^ and Co^3+^ coexisted in all metal-doped samples. Likewise, the high-resolution XPS spectra of Ni 2p, shown in Fig. 6b, exhibit a double peak feature of Ni 2p_3/2_ and Ni 2p_1/2_ spin-orbit components, accompanied by four shake-up satellite peaks.

The peaks at binding energies of 854.1 eV and 872.3 eV are attributed to Ni^2+^, while those at 855.2 eV and 873.4 eV are assigned to Ni species. Additionally, the O 1 s region was analyzed, as illustrated in Fig. 6c. The asymmetric O 1 s peak was deconvoluted into two components: one at ~ 529 eV, corresponding to lattice oxygen (O_L_, O1), and another at ~ 531 eV, associated with surface oxygen vacancies (O_V,_ O_2_)^42^. With Mg and Bi doping, the ions maintained their oxidation states of Co^3+^, Co^2+^, Ni^2+,^ and Ni^3+^, shown in Fig. 6 (c). Furthermore, the O_V_/O_L_ ratio for the Zn-doped (Co_0.9_Ni_0.1_)_3_O_4_ sample was approximately 1.01, which is notably higher than the ratios observed for the Mg- and Bi-doped samples, calculated to be around 0.8 and 0.4, respectively. The higher ratio indicates the existence of many more surface oxygen vacancy sites in the Zn-doped (Co_0.9_Ni_0.1_)_3_O_4_ sample than in the Mg and Bi-doped samples.

**Fig. 7:**
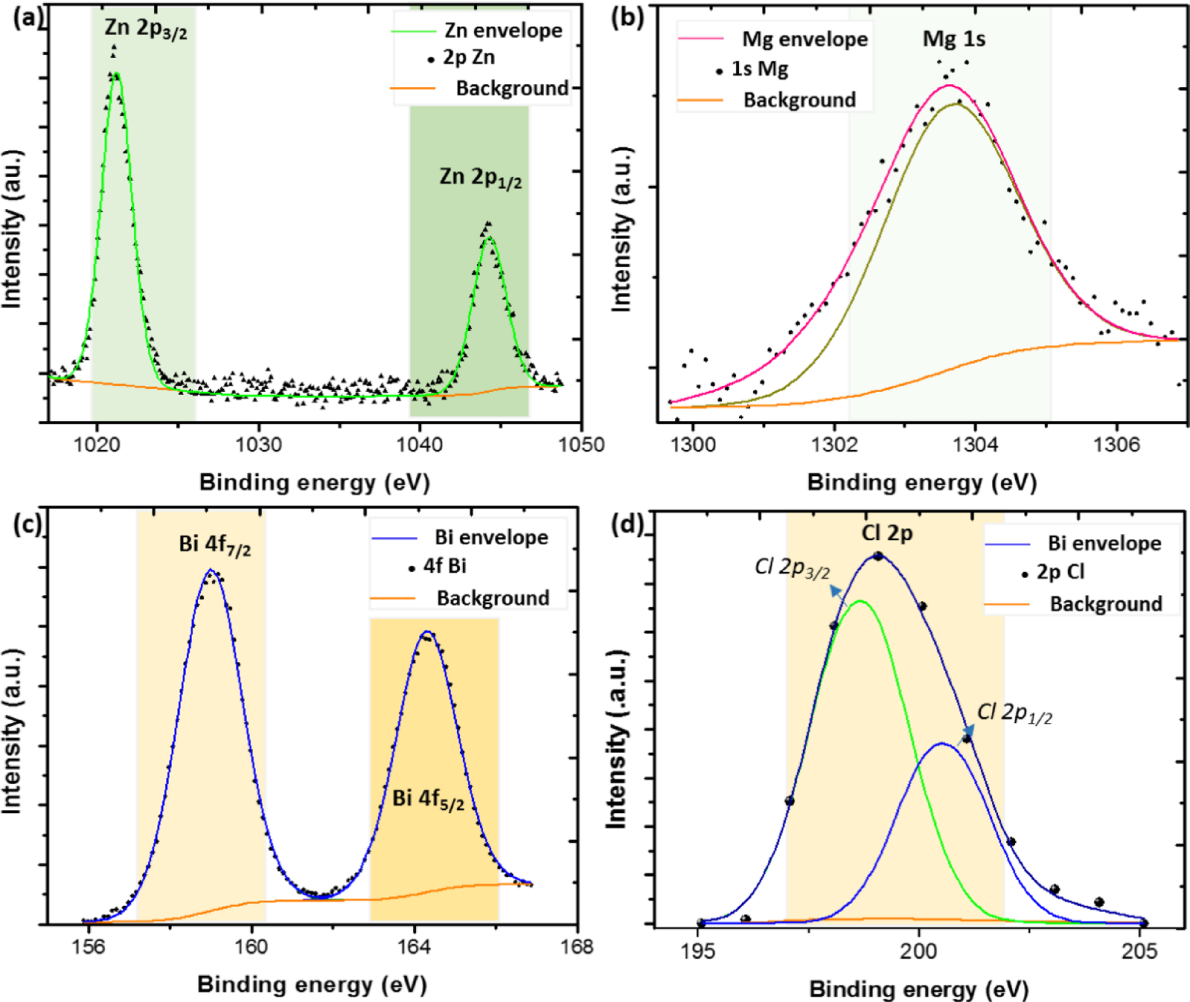
High-resolution XPS spectra of (a) Zn 2p, (b) Mg 1 s, (c) Bi 4f, and (d) Cl 2p, for (Co_0.8_Ni_0.1_ X _0.1_)_3_O_4_ nanoparticles.

The Zn 2p core-level spectrum of the Zn-doped sample, shown in Fig. 7(a), displays two distinct peaks at binding energies of 1022 eV and 1044.5 eV. These correspond to the Zn 2p_3/2_ and Zn 2p_1/2_ spin–orbit components, respectively, and are consistent with the presence of Zn in the + 2 oxidation state, as reported in previous studies^43,44^. Furthermore, the Mg 1 s core-level spectrum of the Mg-doped sample, shown in Fig. 7(b), displays a distinct peak at a binding energy of 1304.5 eV. This peak is indicative of magnesium in the + 2 oxidation state^45^. These findings confirm the successful incorporation of Zn^2+^  and Mg^2+^ ions into their corresponding doped samples. In the case of the Bi-doped sample, the Bi 4f core-level spectrum presented in Fig. 7(c) reveals two well-defined peaks at binding energies of 158.9 eV and 164.2 eV. These correspond to the Bi4f_7/2_ and Bi4f_5/2_ spin-orbit components, respectively, and are characteristic of Bi^3+^ oxidation states^46^. Furthermore, as illustrated in Fig. 7(d), two additional weak peaks appear at approximately 198 eV and 199.6 eV, corresponding to the Cl 2p_3/2_ and Cl 2P_1/2_ components, respectively^31^. The presence of these Cl-related peaks further supports the formation of the BiOCl phase, consistent with the results obtained from XRD and FTIR analyses. Overall, the XPS investigation revealed that the surface composition of the Zn, Mg, and Bi-doped samples includes Co^3+^, Co^2+^, Ni^3+^, Ni^2+^, O^2−^, Zn^2+^, Mg^2+^, Bi^3+,^ and Cl^−^ ions.

### SEM-EDX analysis

**Fig. 8:**
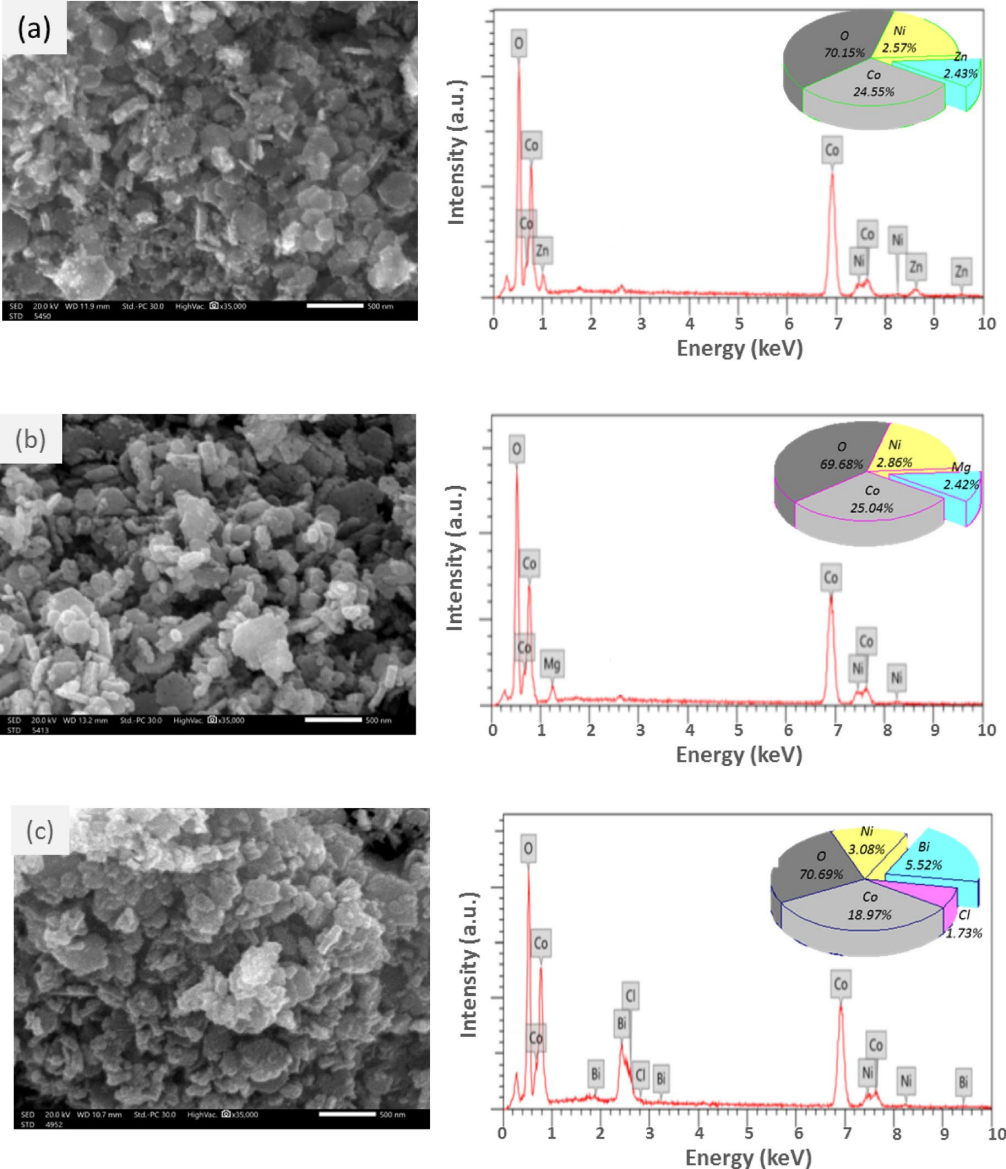
(a-c) SEM images and corresponding EDX elemental analyses of (a) Zn, (b) Mg, and (c) Bi-doped (Co_0.8_Ni_0.1_ X _0.1_)_3_O_4_ nanoparticles.

Figure 8 (a–c) shows the SEM images and their corresponding EDX spectra of Zn, Mg, and Bi-doped (Co_0.8_Ni_0.1_ X _0.1_)_3_O_4_ nanoparticles. All the samples display aggregated particles of different morphologies, including spherical, rod-like, rectangular, and hexagonal, with almost uniform distribution. Additionally, a few larger lumps can be seen, particularly for the Bi-doped (Co_0.9_Ni_0.1_)_3_O_4_ sample. These larger aggregates may result from particles stacking on top of each other or the growth of new particles, like BiOCl. Upon closer inspection, it appears that the stacked particles are the smallest in size, which could be due to their significantly higher surface energy, leading to agglomeration. Another possible explanation is incomplete or irregular crystal formation, where seed particles may have failed to grow in proper orientation, resulting in an irregular or highly agglomerated structure^47^. The chemical composition and element atomic percentage (At%) were recorded by EDX spectroscopy as shown in Fig. 8(a-c). The EDX spectra of metal-doped samples suggest a chemical composition that consists mainly of Co and O, the major peaks of synthesized nanoparticles. Moreover, some minor peaks are attributed to Ni, Zn, Mg, and Bi as expected, evidencing the compositional purity of the synthesized samples. The surface atomic percentage (At% %) of the constituent elements in respective samples is presented in the insets of Fig. 8(a-c). However, the presence of small amounts of Cl in the Bi-doped sample (Fig. 8c) confirms the phase formation of BiOCl, which is consistent with the XRD, FTIR, and XPS analyses.

### TEM, HRTEM, and SAED analysis

**Fig. 9:**
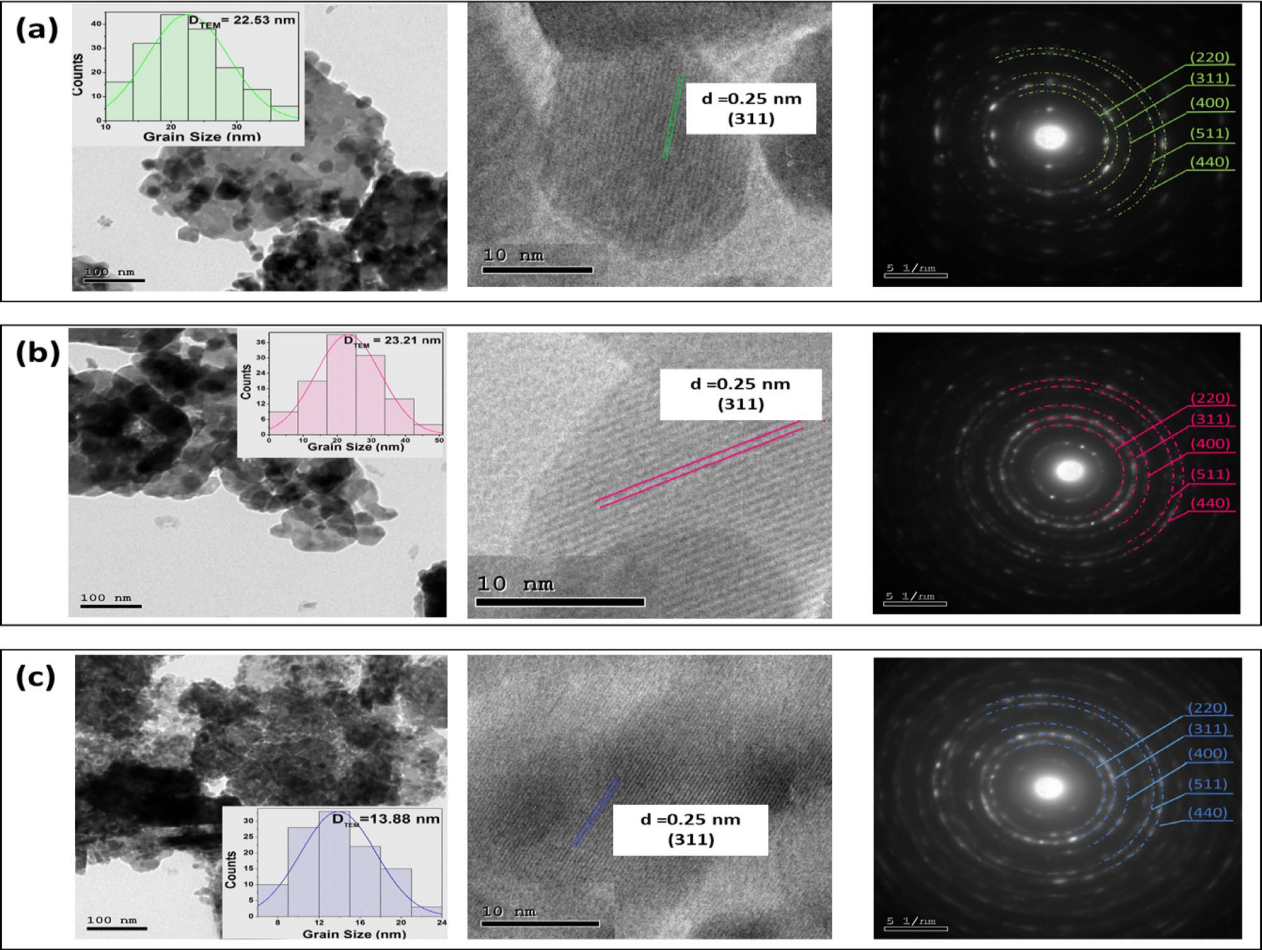
TEM (inset: histogram of particle size distribution), SEAD, and HRTEM images of (a) Zn, (b) Mg, and (c) Bi-doped (Co_0.8_Ni_0.1_ X _0.1_)_3_O_4_ nanoparticles, respectively.

The TEM micrographs of Zn, Mg, and Bi-doped (Co_0.8_Ni_0.1_ X _0.1_)_3_O_4_ samples are displayed in Fig. 9 (a-c), respectively. For Zn and Mg-doping, the particles were agglomerated and larger, with distinct nanostructures. The particles are mostly spherical, with some hexagonal and cubic-shaped nanoparticles. With Bi-doped (Co_0.9_Ni_0.1_)_3_O_4_, it is observed that spherical-shaped particles were highly agglomerated with some larger particles. For the particle size analysis, ImageJ software (Version 1.53t)^48^, was used to generate particle size distribution histograms, shown in the insets of TEM images. The size obtained from the TEM images ranged from ~ 13 to ~ 23 nm, which correlated well with the XRD crystalline size. The estimated particle size for Zn-doping is 22.5 nm, Mg-doping is 23.21 nm, and the minimum value is 13.88 nm for Bi-doping. These values are larger than the crystallite size measured from XRD, which can be attributed to the formation of polycrystalline aggregates. This aggregation occurs due to inter-particle magnetic interactions, Van der Waals forces, and the high surface energy between the oxide nanoparticles. These factors promote the particles to cluster together, resulting in larger observed particle sizes compared to the individual crystallites^49^.

For further analysis, the HRTEM images of Zn, Mg, and Bi-doped (Co_0.8_Ni_0.1_ X _0.1_)_3_O_4_ nanoparticles, presented in Fig. 9 (a–c), reveal distinct lattice fringes with a d-spacing of approximately 0.25 nm, which corresponds to the (311) plane of the spinel cubic structure. Additionally, no secondary phase formation was observed in the HRTEM images.

**Fig. 10:**
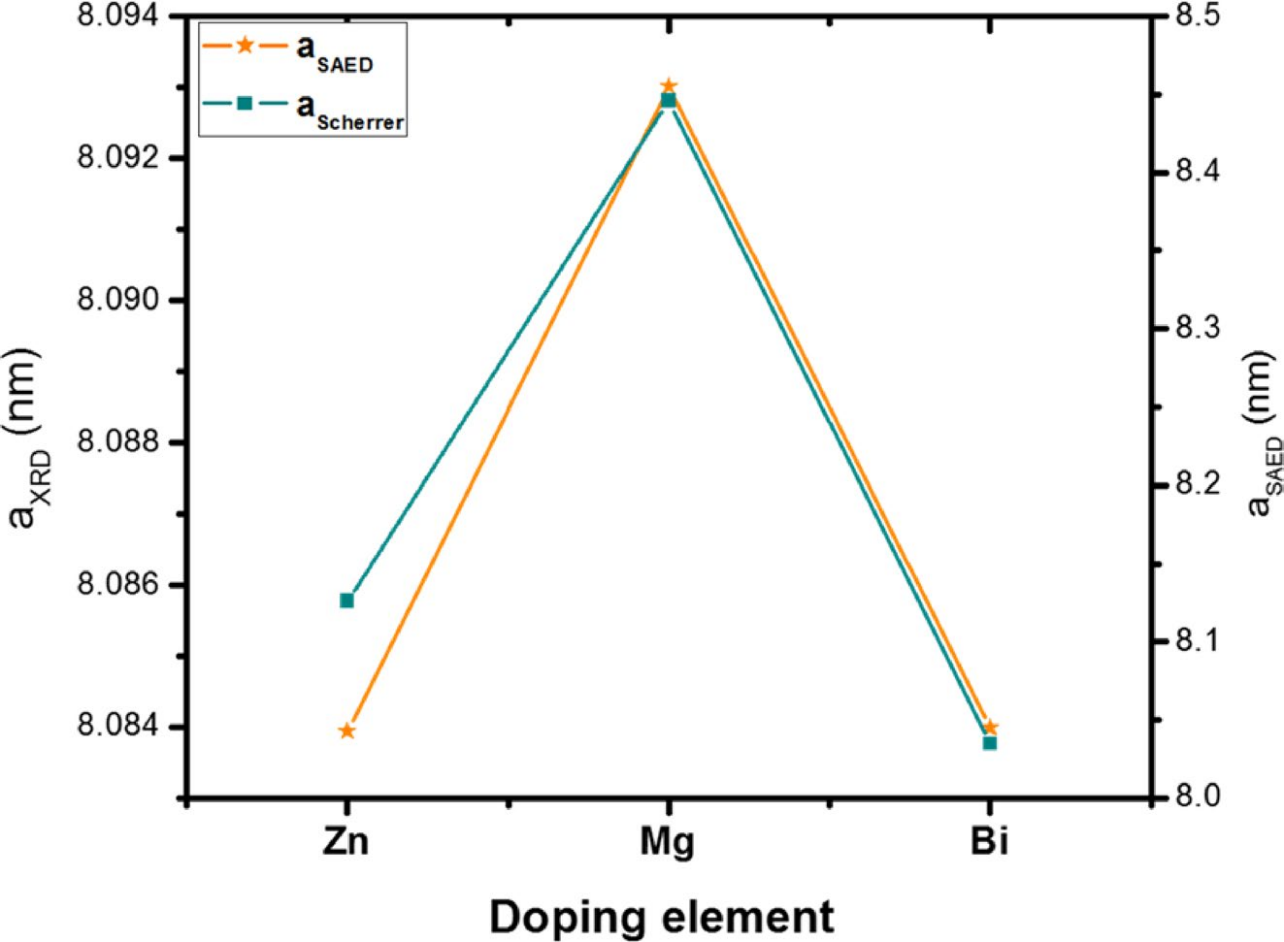
Comparison of lattice parameters from XRD and SAED techniques of (Co_0.8_Ni_0.1_ X _0.1_)_3_O_4_ nanoparticles.

The corresponding SAED patterns of Zn, Mg, and Bi-doped (Co_0.8_Ni_0.1_ X _0.1_)_3_O_4_ nanoparticles are included in Fig. 9 (a-c). It displays a well-defined arrangement characterized by distinct one-centered diffraction rings with varying radii. These diffraction rings correspond to the lattice planes of the cubic structure of Zn, Mg, and Bi-doped (Co_0.8_Ni_0.1_ X _0.1_)_3_O_4_, specifically (220), (311), (400), (511), and (440). The calculated lattice parameters obtained from the SAED pattern are in close agreement with those derived from the XRD pattern, as shown in Fig. 10. Although the nanoparticles obtained were small, complete uniformity in particle dispersion was not achieved. This lack of uniformity resulted in the broadening of the diffraction rings, which exhibited multiple diffraction spots, suggesting the presence of a polycrystalline structure.

### N_2_ adsorption-desorption technique

**Fig. 11:**
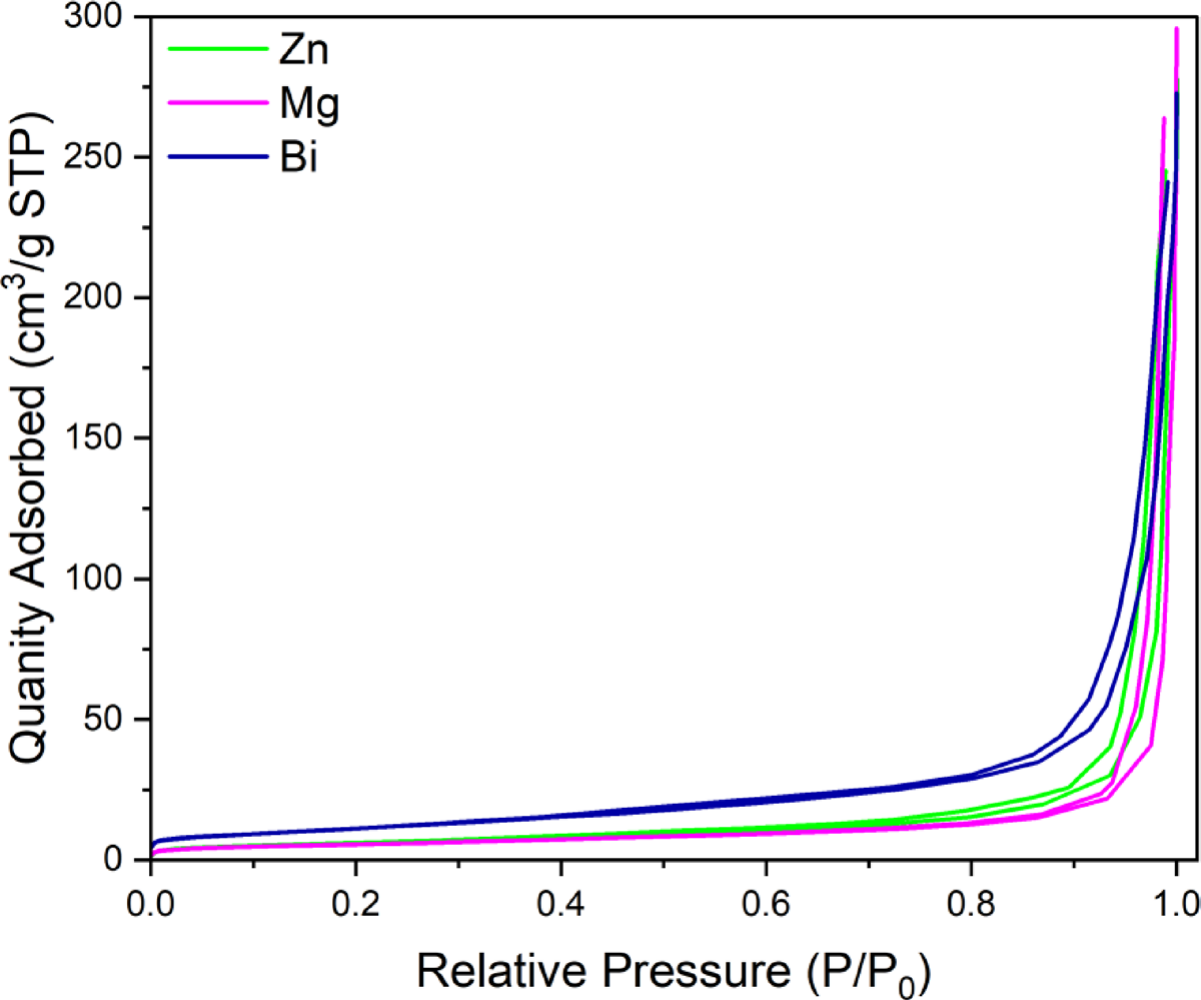
N_2_ adsorption-desorption isotherm for Zn-doped, Mg-doped, and Bi-doped (Co_0.8_Ni_0.1_ X _0.1_)_3_O_4_ nanoparticles.

Brunauer-Emmett-Teller (BET) and Barrett-Joyner-Halenda (BJH) analyses were utilized to determine the textural properties of (Co_0.8_Ni_0.1_Zn_0.1_)_3_O_4_, (Co_0.8_Ni_0.1_Mg_0.1_)_3_O_4_, and (Co_0.8_Ni_0.1_Bi_0.1_)_3_O_4_ nanoparticles. The N_2_ adsorption-desorption plot, shown in Fig. 11, illustrates a type IV isotherm with H3 hysteresis loop according to IUPAC classifications^50^. Table 2 lists the surface area (S_BET_), the pore volume, and the pore diameter for the prepared samples. Among them, the highest surface area was detected for the Bi-doped sample, reaching 41.23 m^2^.g^−1^, compared to the Zn- and Mg-doped samples, which showed 21.17 and 19.7 m^2^.g^−1^, respectively. This high surface area in the Bi-doped sample may be attributed to the crystal distortion and inhibited particle growth associated with the formation of the BiOCl secondary phase, as discussed earlier in the XRD analysis section. Additionally, the Zn-doped nanoparticles exhibited the highest pore volume and diameter. The obtained pore diameters for all samples confirm their mesoporous nature.

**Table 2:** Textural properties of (Co_0.8_Ni_0.1_ X _0.1_)_3_O_4_ nanoparticles.

Nanoparticles	S_BET_ (m^2^.g^−1^)	Pore Volume (cm^3^.g^−1^)	Pore diameter (nm)
(Co_0.8_Ni_0.1_Zn_0.1_)_3_O_4_	21.17	0.34	57.79
(Co_0.8_Ni_0.1_Mg_0.1_)_3_O_4_	19.7	0.27	49.4
(Co_0.8_Ni_0.1_Bi_0.1_)_3_O_4_	41.23	0.34	35.75

### Raman spectroscopy

**Fig. 12:**
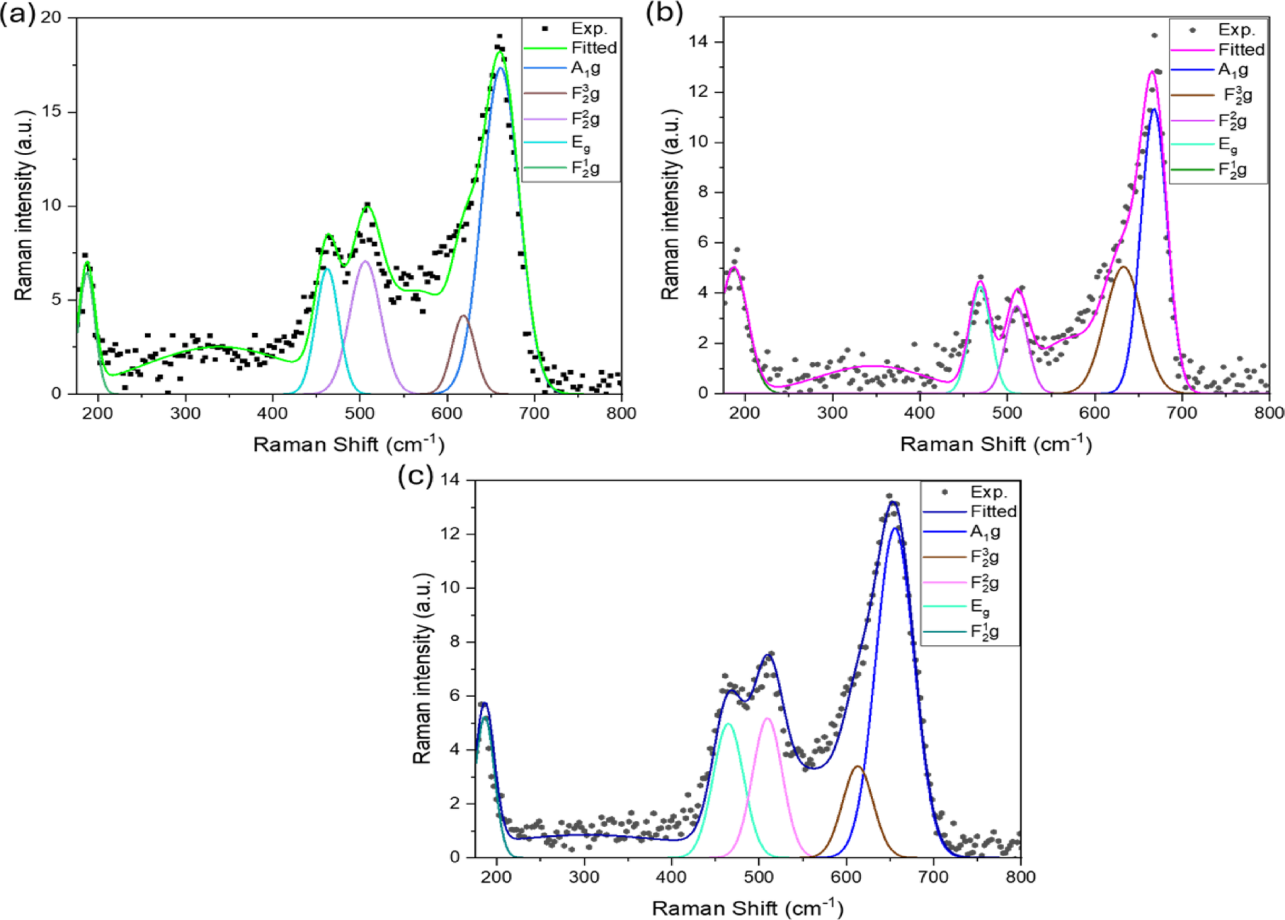
**(a-c)** Deconvolution of Raman spectra of Zn, Mg, and Bi-doped (Co_0.8_Ni_0.1_ X _0.1_)_3_O_4_ nanoparticles, respectively.

The room temperature Raman spectra were performed to detect any trace amount of secondary phases in the metal-doped (Co_0.8_Ni_0.1_ X _0.1_)_3_O_4_ nanoparticles. Figure 12(a-c) shows the deconvoluted Raman spectra of Zn, Mg, and Bi-doped (Co_0.8_Ni_0.1_ X _0.1_)_3_O_4_ nanoparticles, respectively. For all samples, the Raman spectra are nearly identical. Five distinct Raman active modes were observed at approximately 661, 621, 509, 465, and 187 cm^−1^, characteristic of the cubic spinel structure of Co_3_O_4_^51^. These peaks correspond to A_1g_, $\:{F}_{2g}^{3}$, $\:{F}_{2g}^{2}$, *E*_*g*_, and $\:{F}_{2g}^{1}$ vibrational modes of Co_3_O_4_, respectively, as labeled in Fig. 12(a-c). The phonon symmetries of Raman peaks result from the lattice vibrations of the spinel structure, where Co^2+^ and Co^3+^ cations occupy tetrahedral and octahedral sites, respectively, in the cubic lattice^52^. The A_1g_ mode at ⁓ 661 cm^−1^, the highest frequency peak, associated with Co^3+^-O stretching in the octahedral sites (CoO_6_)^53^. The $\:{F}_{2g}^{1}$ and $\:{F}_{2g}^{2}$ modes are particularly sensitive to the cation occupancy in the tetrahedral sites, while the *E*_*g*_ mode arises due to the combined vibrations of the tetrahedral site and octahedral oxygen motions^54–56^. Hence, these findings confirm the successful formation of the spinel-phase metal-doped (Co_0.8_Ni_0.1_ X _0.1_)_3_O_4_ nanoparticles.

Additionally, to further investigate the effect of metal doping on the lattice distortion in the spinel structure of Co_3_O_4_. Raman peak broadening and wavenumber shift are spectral features commonly affected by factors such as crystallite size and surface-induced stress or strain^57^. Table 3 presents the estimated fitting parameters, such as peak position and FWHM of the deconvoluted Raman peaks using Lorentzian function for metal-doped (Co_0.8_Ni_0.1_ X _0.1_)_3_O_4_ nanoparticles. Compared to Zn and Mg-doped samples, the most sensitive and prominent peak A_1_g of the Bi-doped sample exhibits a slight red shift toward lower wavenumbers along with an increase in FWHM, indicating the formation of a more defective structure. This observation is consistent with a reduction in crystallite size in the Bi-doped sample and the appearance of a secondary BiOCl phase, as revealed by XRD and FTIR analyses. The presence of this secondary phase likely contributes to lattice contraction and increased lattice distortion, as further confirmed by XRD results.

**Table 3:** Peak position and FHWM of deconvoluted Raman spectra of zn, mg, and Bi-doped (Co_0.8_Ni_0.1_ X _0.1_)_3_O_4_ samples.

Raman band	Zn-doped		Mg-doped		Bi-doped	
	Position (cm^−1^)	FWHM	Position (cm^−1^)	FWHM	Position (cm^−1^)	FWHM
$\:{{F}}_{2{g}}^{1}$	187.0	21.6	187.0	37.42	186.1	25.8
E_g_	462.4	30.8	468.7	28.6	464.9	41.0
$\:{{F}}_{2{g}}^{2}$	506.0	41.8	510.6	29.6	509.7	40.0
$\:{{F}}_{2{g}}^{3}$	618.5	30.8	632.7	49.6	613.1	41.2
A_1_g	661.1	49.9	667.7	35.2	655.6	51.6

### UV–Vis spectroscopy

**Fig. 13:**
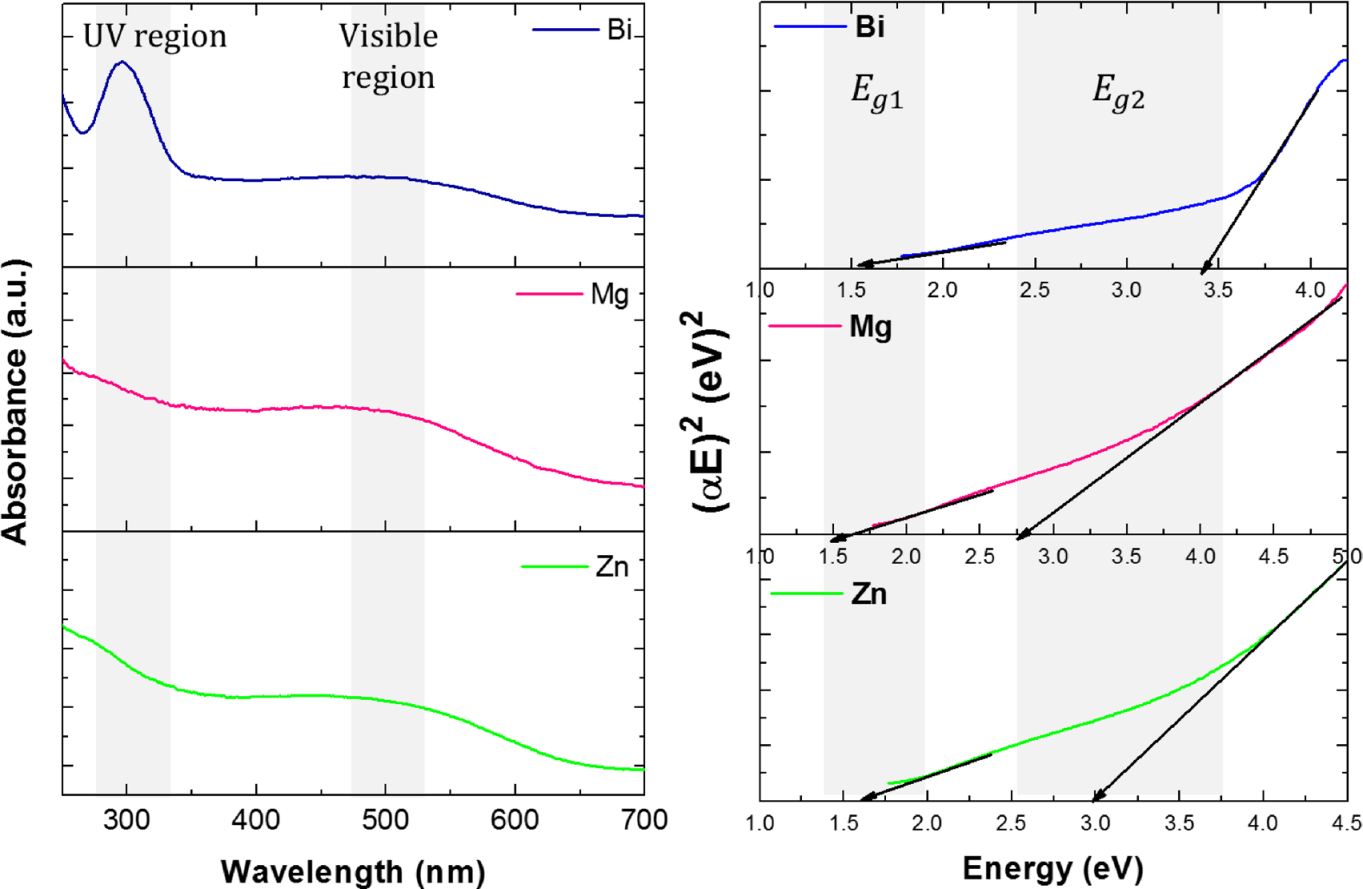
(a) UV-Vis absorption spectra, and (b) Tauc’s plot for the energy band gap of Zn, Mg, and Bi-doped (Co_0.8_Ni_0.1_X _0.1_)_3_O_4_ nanoparticles.

**Fig. 14:**
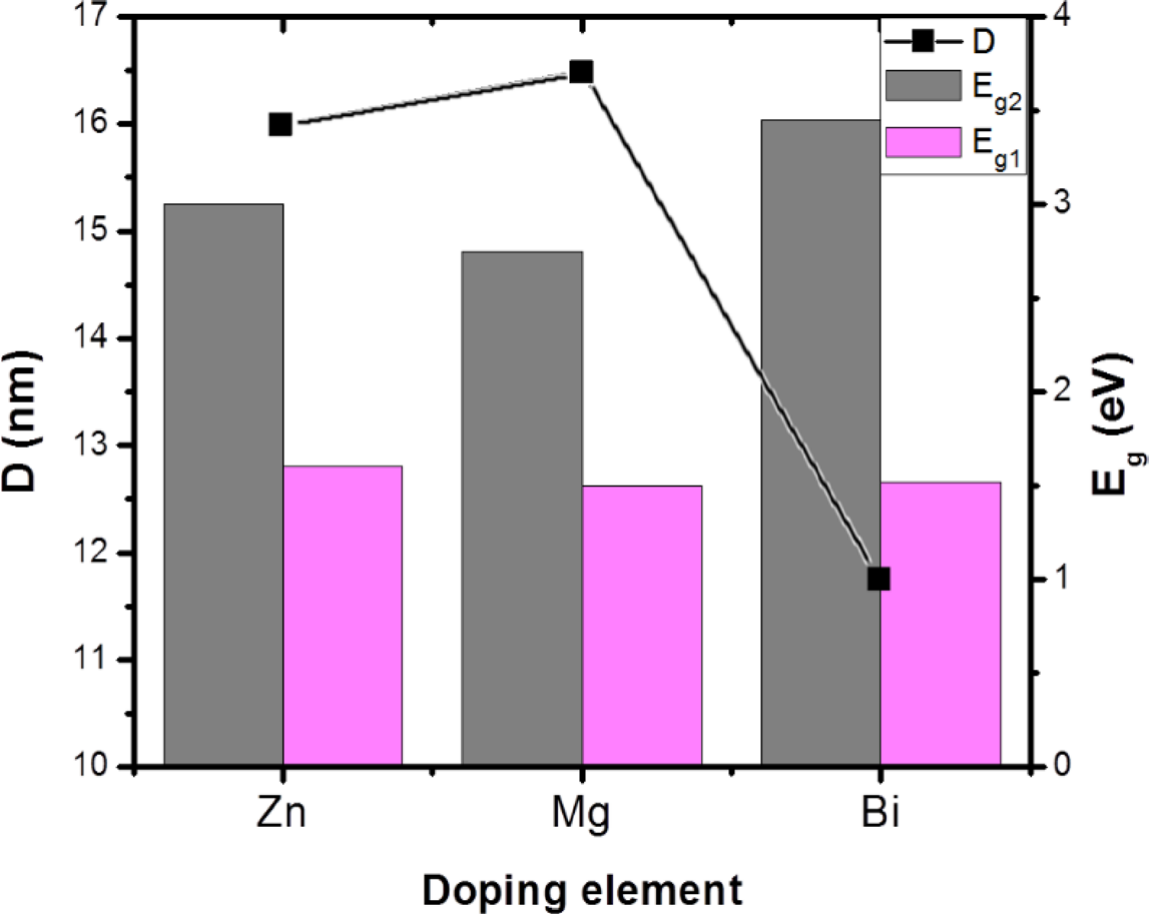
The dependence of band gap energies on crystallite size of Zn, Mg, and Bi-doped (Co_0.8_Ni_0.1_ X _0.1_)_3_O_4_ nanoparticles.

It is well known that the size, shape, and dimensions of materials strongly influence the optical band gap of p-type Co_3_O_4_. Figure 13(a) shows the UV-Vis absorption spectra of the X = Zn, Mg, and Bi-doped (Co_0.8_Ni_0.1_ X _0.1_)_3_O_4_ nanoparticles. The curves exhibit two different absorption bands, indicating the persistence of two distinct oxidation states for cobalt ions (Co^3+^ and Co^2+^). The absorption band centered at the UV region is due to the charge transfer transition of O^2−^ to Co^2+^ (valence to conduction band excitation), while the band centered at the visible region is ascribed to O^2−^ to Co^3+^ transition (Co^3+^ level placed below the conduction band)^58^. These transitions confirmed the formation of a spinel cobalt oxide structure. Furthermore, the metal-doped samples exhibited a blue or red shift depending on the kind and amount of the dopant ions. For energy gap (*E*_*g*_) estimation of the prepared doped (Co_0.9_Ni_0.1_)_3_O_4_ samples, the well-known Tauc’s relation was used^59^:

6 $$\:\alpha\:h\nu\:\:=\:A{(h\nu\:-{E}_{g})}^{n},$$

where ($\:\alpha\:)$is the absorption coefficient, A is a constant, $\:h\nu\:\:$photon energy, and n is 1/2 for the direct band gap of the semiconductors. Graphically, the linear extrapolation of (*αhν*)^2^ along the y-axis versus $\:h\nu\:$ on the x-axis intersects at *E*_*g*_, as shown in Fig. 13(b). As can be seen, the curve can be linearly fitted with two straight line portions, indicating the existence of two direct band gaps, corresponding to $\:{E}_{g1}$ and $\:{E}_{g2}$ (with *E*_*g1*_> E_g2_). The estimated values of band gaps and crystallite size of metal-doped samples are shown in Fig. 14. It is observed that the band gap energy for the Bi-doped sample is higher compared to those of Zn, and Mg-doped samples. This behavior can be attributed to the quantum confinement effect, where the band gap energy increases as the crystallite size decreases, especially in low-dimensional systems. Therefore, the band gap energy is inversely proportional to the square of the crystallite size (*1/D²*), due to quantum confinement^60^. Further, the increase of energy gap values with the Bi-doped sample may be due to the presence of the second phase (BiOCl) of a wide band gap 3.05–3.55 eV^61^.

The lower band gap values observed in Zn and Mg-doped samples are attributed to the introduction of electronic states within the bulk band gap, associated with the dopant ions and the formation of crystal defects. This observation is supported by Lakehal et al.^62^, reporting that the band gaps of Co_3_O_4_ thin films doped with transition metals (TM = Ni, Mn, and Cu) at a doping ratio of 9 wt.% decreased with doping. Similarly, in the case of Cu_x_Co_3-x_O_4_ nanoparticles, a reduction in band gap at higher Cu content has been linked to defect formation within the crystal structure^63^. It is worth mentioning that the $\:{E}_{g1}$ and $\:{E}_{g2}\:$values of metal-doped (Co_0.9_Ni_0.1_)_3_O_4_ nanoparticles were larger than those of bulk Co_3_O_4_ in the range 1.88–2.13 eV and 1.50–1.52 eV, respectively^24,46^. This could be due to the metal-doped samples at the nanoscale.

### Photoluminescence (PL) analysis

**Fig. 15:**
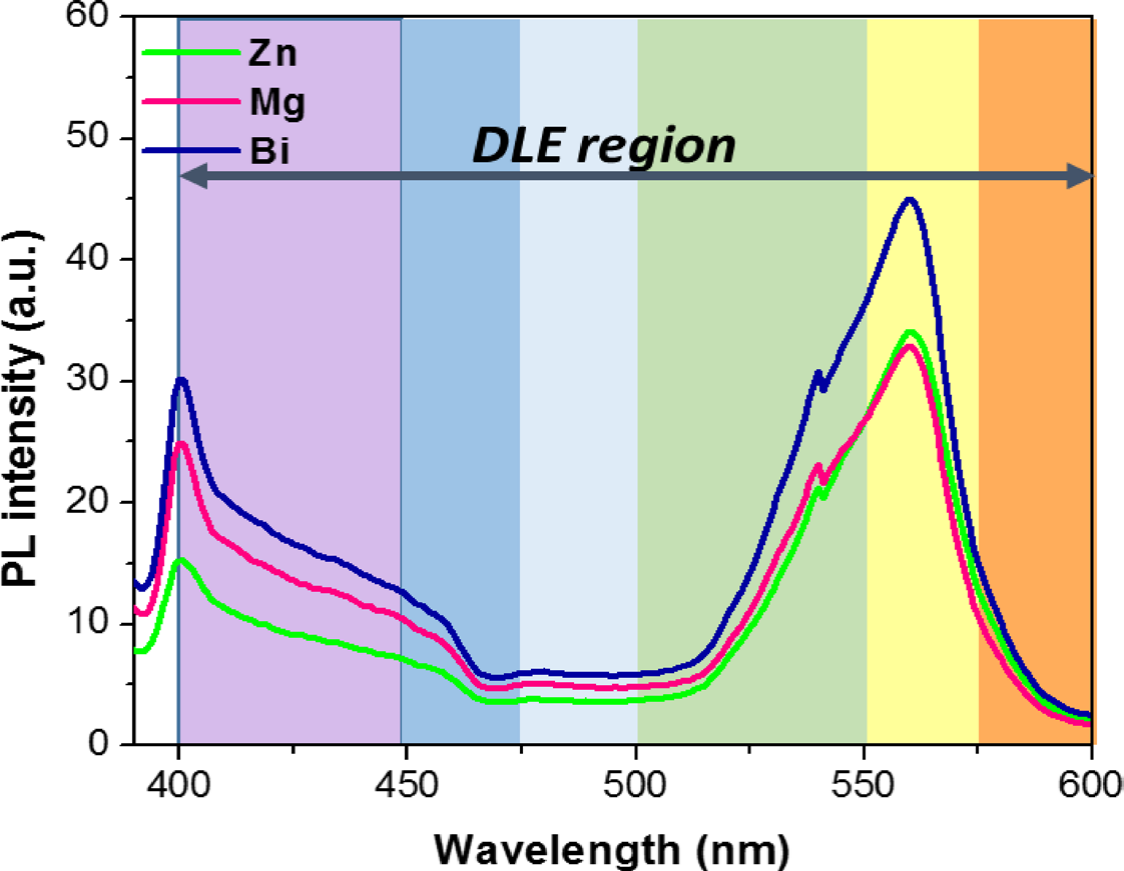
PL spectra of Zn, Mg, and Bi-doped (Co_0.8_Ni_0.1_X _0.1_)_3_O_4_ nanoparticles.

The photoluminescence (PL) emission spectra of semiconductors originate from the recombination of electron-hole pairs that have been photoexcited and provide insights into the transfer, migration, and separation of generated charge carriers. The measured PL spectra at room temperature Zn, Mg, and Bi-doped (Co_0.8_Ni_0.1_ X _0.1_)_3_O_4_ at an excitation wavelength of 250 nm are displayed in Fig. 15. The PL spectra of all samples revealed two maxima in the range 340–650 nm. The first is the deeper emission around 400 nm, referring to the violet emission. The second peak, in the green region, ranges around 560 nm with a small shoulder peak of around 539 nm, caused by the defects and oxygen vacancies (V_o_) within the nanoparticles. These defects stabilize the Co_3_O_4_ cubic spinel structure^64^. It is worth mentioning that the blue band is ascribed to the O^2−^– Co^2+^ charge transfer process, whereas the green emission is related to the O^2−^– Co^3+^ charge transfer^64^. However, the reduction in the intensity of PL emission peaks resulted from the slow/delayed recombination rate of photogenerated electrons and holes^65^. As depicted in Fig. 15 (Co_0.8_Ni_0.1_Zn_0.1_)_3_O_4_ nanoparticles presented a weaker intensity emission peak compared to Mg and Bi-doped (Co_0.8_Ni_0.1_X_0.1_)_3_O_4_ nanoparticles, indicating a lower rate of electron-hole recombination. It is evident from previous reports that irradiative transitions between shallow donors (related to oxygen vacancies) produce strong UV emissions and suppressed visible emission, confirming the crystal quality of (Co_0.8_Ni_0.1_Zn_0.1_)_3_O_4_ nanoparticles.

### Adsorption activity

#### Influence of contact time

**Fig. 16:**
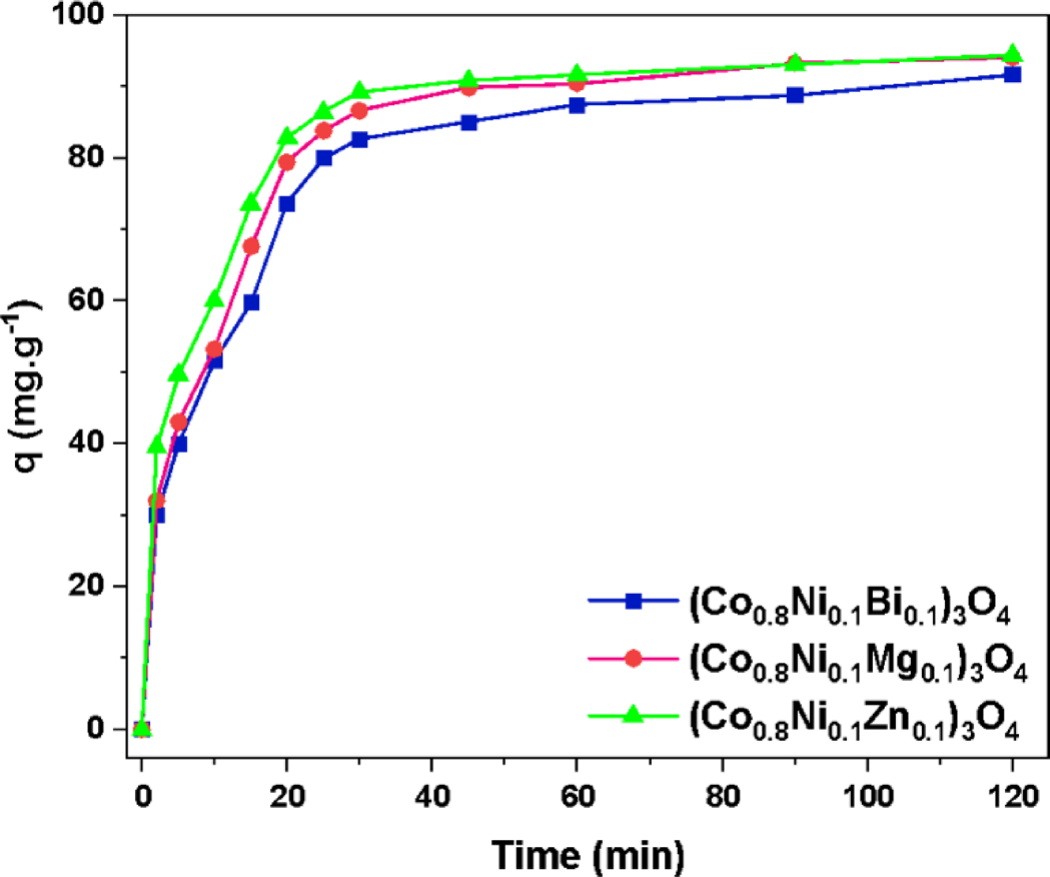
Influence of contact time on the adsorption capacity (q_t_) evaluated in the presence of Zn, Mg, and Bi-doped (Co_0.8_Ni_0.1_ X _0.1_)_3_O_4_ nanoparticles.

The adsorption efficiency of Zn, Mg, and Bi-doped (Co_0.8_Ni_0.1_ X _0.1_)_3_O_4_ nanoparticles was assessed for the removal of methylene blue dye. The impact of contact time on the adsorption capacity (q_t_) of methylene blue was investigated, and the results are presented in Fig. 16. As the contact time increases up to 30 min, the adsorption capacity rises significantly. However, extending the contact time beyond 30 min results in only a slight increase, likely due to the saturation of available binding sites^66^. As a result, additional adsorption becomes negligible, and equilibrium is reached after 30 min. After a contact time of 30 min, the adsorption capacity is determined to be 82.6, 86.6, and 89.2 mg.g^−1^ in the presence of (Co_0.8_Ni_0.1_Bi_0.1_)_3_O_4_, (Co_0.8_Ni_0.1_Mg_0.1_)_3_O_4_, and (Co_0.8_Ni_0.1_Zn_0.1_)_3_O_4_ nanoparticles, respectively. Furthermore, the maximum adsorption capacity was achieved after 120 min. Consequently, the adsorption capacity is to be 91.6, 94, and 94.4 mg.g^−1^ in the presence of (Co_0.8_Ni_0.1_Bi_0.1_)_3_O_4_, (Co_0.8_Ni_0.1_Mg_0.1_)_3_O_4_, and (Co_0.8_Ni_0.1_Zn_0.1_)_3_O_4_ nanoparticles, respectively. Therefore, (Co_0.8_Ni_0.1_Zn_0.1_)_3_O_4_ nanoparticles exhibit the highest adsorption capacity among the investigated samples for removing methylene blue. This is attributed to the high pore diameter and volume revealed for Zn-doped nanoparticles. As a result, larger pores offer increased space for adsorbate molecules to be trapped and held, thereby enhancing the overall adsorption performance^67^. El-Dossoki et al.^68^, reported that the maximum adsorption capacity revealed by Co_3_O_4_ nanoparticles was 27.2 mg.g^−1^. Thus, superior adsorption capacity is obtained by nanoparticles prepared in this study, compared to the literature, as listed in Table 4.

It is important to note that the (Co_0.8_Ni_0.1_Bi_0.1_)_3_O_4_ sample exhibited a secondary BiOCl phase, as confirmed by XRD, FTIR, and XPS analyses. BiOCl, a layered semiconductor, is known for its large surface area and distinctive electronic properties^69^. This explains the high BET surface area of the Bi-doped sample (41.23 m²/g), compared to the Zn- and Mg-doped samples. Despite this advantage, the adsorption capacity of the Bi-doped sample was slightly lower than the others. This is likely due to its lower oxygen vacancy concentration (O_V_/O_L_ ratio ~ 0.4), as revealed by XPS, which plays a critical role in dye molecule interactions. Additionally, the BiOCl phase alters the surface charge properties of the nanoparticles, which was reflected in the pH-dependent adsorption behavior (discussed in details below). In summary, BiOCl contributes to increased surface area and favorable acidic adsorption, but its lower oxygen vacancy density and reduced surface reactivity may limit its adsorption performance under neutral or basic conditions.

**Table 4:** Literature review for various adsorbents on MB dye adsorption.

Absorbent NPs	Adsorbent Dosage	MB concentration (mg.L^−1^)	Adsorption Capacity (mg.g^−1^)	Ref.
Co_3_O_4_	-	10	27.2	^68^
CuO	-	50	2.497	^70^
ZnO	0.2 g/L	40	9.6	^71^
ZnO	0.2 mg/mL	90	33.73	^72^
Graphene oxide/ZnTiO_3_/TiO_2_	100 mg	10	78	^73^
(Co_0.8_Ni_0.1_Mg_0.1_)_3_O_4_ (Co_0.8_Ni_0.1_Bi_0.1_)_3_O_4_ (Co_0.8_Ni_0.1_Zn_0.1_)_3_O_4_	50	50	82.6 86.6 89.2	This study

#### Influence of initial dye concentration

**Fig. 17:**
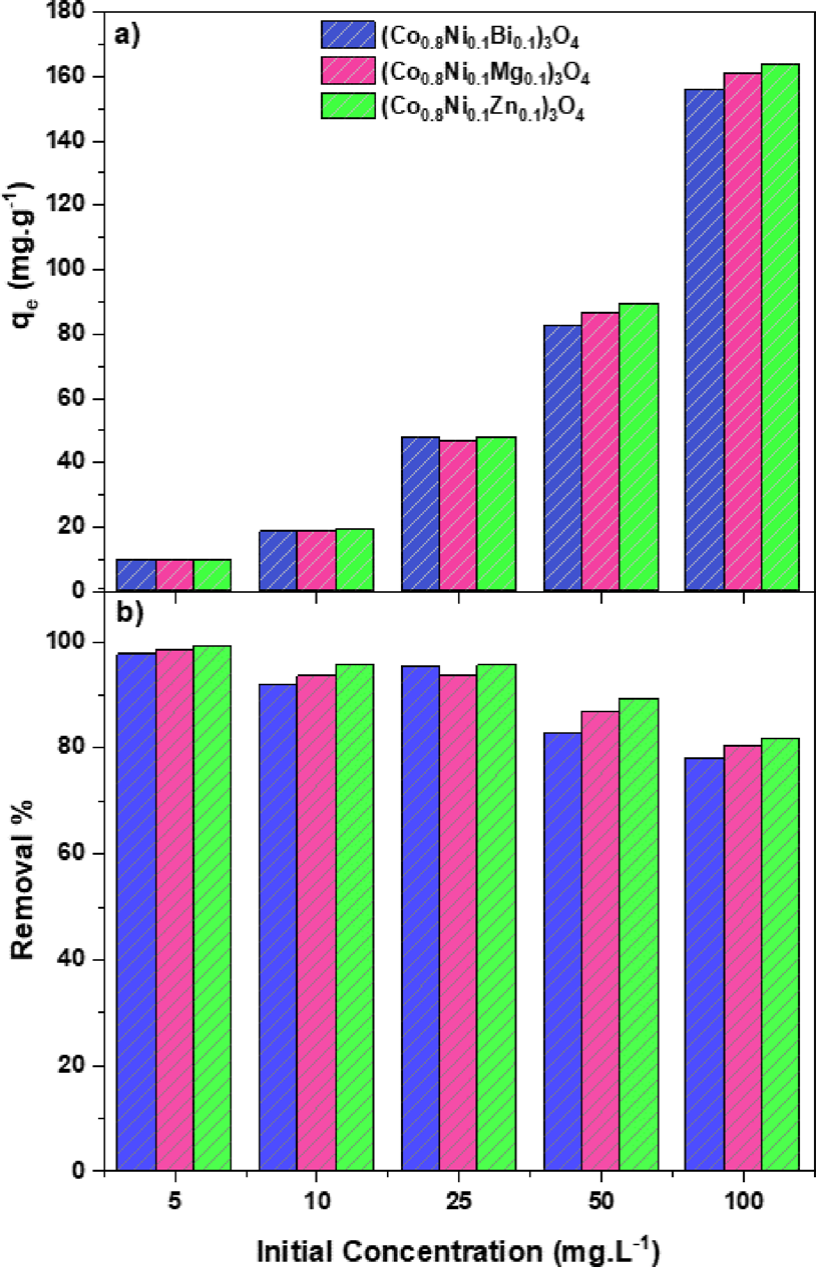
Impact of initial dye concentration on the (a) adsorption capacity and (b) removal% in the presence of Zn, Mg, and Bi-doped (Co_0.8_Ni_0.1_ X _0.1_)_3_O_4_ nanoparticles.

The adsorption experiments were conducted using methylene blue solutions with initial concentrations of 5, 10, 25, 50, and 100 mg.L^−1^ to investigate the effect of initial dye concentration on removal efficiency and adsorption capacity. As the initial concentration rises, there is a corresponding decrease in the removal percentage after a contact time of 30 min, as depicted in Fig. 17 (a). It is worth mentioning that 99%, 98.4%, and 97.6% of 5 mg.L^−1^ methylene blue solution is adsorbed onto (Co_0.8_Ni_0.1_ X _0.1_)_3_O_4_ nanoparticles doped with Zn, Mg, and Bi, respectively. Furthermore, as the initial concentration increases to 100 mg/L, the removal efficiency of (Co_0.8_Ni_0.1_ X _0.1_)_3_O_4_ nanoparticles doped with Zn, Mg, and Bi decreases to 81.8%, 80.3%, and 77.9%, respectively. The observed phenomenon is attributed to the greater accessibility of active sites on the nanoparticles at lower concentrations, facilitating the binding of methylene blue molecules. With an increase in the initial concentration, these active sites become saturated, reducing the removal percentage^74^.

Conversely, the adsorption capacity increases with increasing initial concentration, as illustrated in Fig. 17 (b). The adsorption capacity, determined by using 5 mg.L^−1^ methylene blue solution, is 9.9, 9.84, and 9.76 mg.g^−1^ in the presence of (Co_0.8_Ni_0.1_Zn_0.1_)_3_O_4_, (Co_0.8_Ni_0.1_Mg_0.1_)_3_O_4_, and (Co_0.8_Ni_0.1_Bi_0.1_)_3_O_4_ nanoparticles, respectively. However, adsorption capacity increases with the initial concentration to 100 mg.L^−1^ to reach 163.6, 160.6, and 155.8 mg.g^−1^ in the presence of (Co_0.8_Ni_0.1_Zn_0.1_)_3_O_4_, (Co_0.8_Ni_0.1_Mg_0.1_)_3_O_4_, and (Co_0.8_Ni_0.1_Bi_0.1_)_3_O_4_ nanoparticles, respectively. Following a similar trend to the removal percentage, (Co_0.8_Ni_0.1_Zn_0.1_)_3_O_4_ nanoparticles exhibit the maximum adsorption capacity among the synthesized adsorbents. Notably, at elevated dye concentrations, the mass transfer driving force becomes more pronounced, promoting the migration of dye molecules from the liquid phase to the surface of the solid adsorbent^75^. This phenomenon serves as the primary factor contributing to the enhanced adsorption capacity observed at higher initial dye concentrations.

#### Influence of pH

**Fig. 18:**
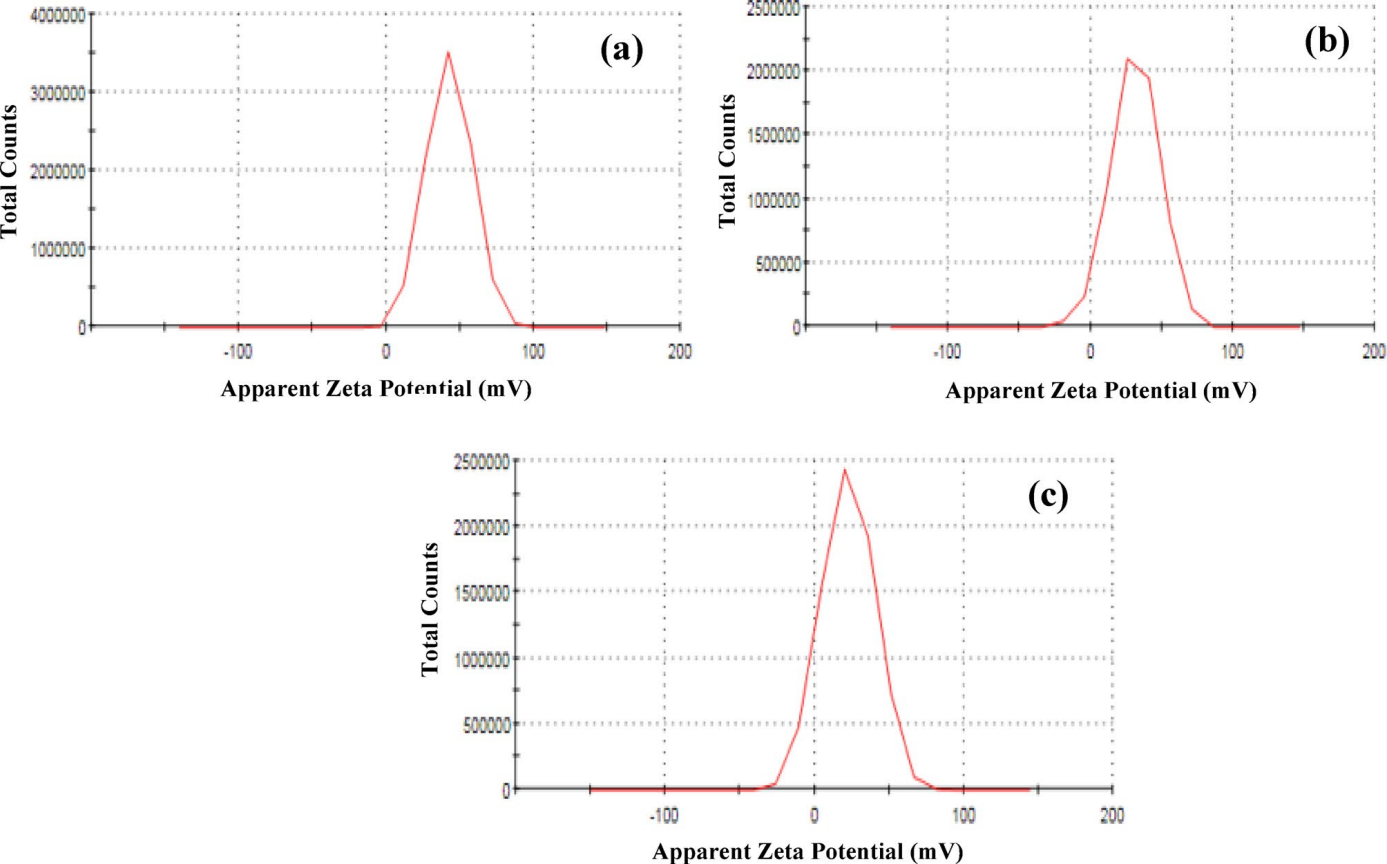
(a-c) Zeta potential measurement of Zn, Mg, and Bi-doped (Co_0.8_Ni_0.1_ X _0.1_)_3_O_4_ nanoparticles in aqueous solution, respectively.

The zeta potential of an adsorbent is a critical indicator of its surface charge, influencing both the stability of colloidal suspensions and the adsorption interactions with charged species, such as dye molecules in solution. As shown in Fig. 18, the zeta potential values of (Co_0.8_Ni_0.1_Zn_0.1_)_3_O_4_(+ 42.6 mV), (Co_0.8_Ni_0.1_Mg_0.1_)_3_O_4_, (+ 31.8 mV), and (Co_0.8_Ni_0.1_Bi_0.1_)_3_O_4_ (+ 22.7 mV) were measured under specific conditions. All values indicate a positively charged surface, suggesting a natural electrostatic repulsion toward methylene blue (MB), a cationic dye, especially under acidic or neutral conditions. However, the adsorption behavior of MB is strongly pH-dependent, as pH affects both the surface charge of the adsorbent and the ionization state of the dye. At low pH, the surface of the metal oxides tends to be more positively charged due to protonation of surface hydroxyl groups, thereby increasing electrostatic repulsion with MB and decreasing adsorption efficiency. As the pH increases, surface deprotonation occurs, leading to a reduction in positive surface charge, and in some cases, a shift toward neutral or even negative zeta potential, depending on the material and its point of zero charge (pHpzc). This shift significantly enhances the electrostatic attraction between the adsorbent and the cationic MB molecules, thus promoting adsorption.

**Fig. 19:**
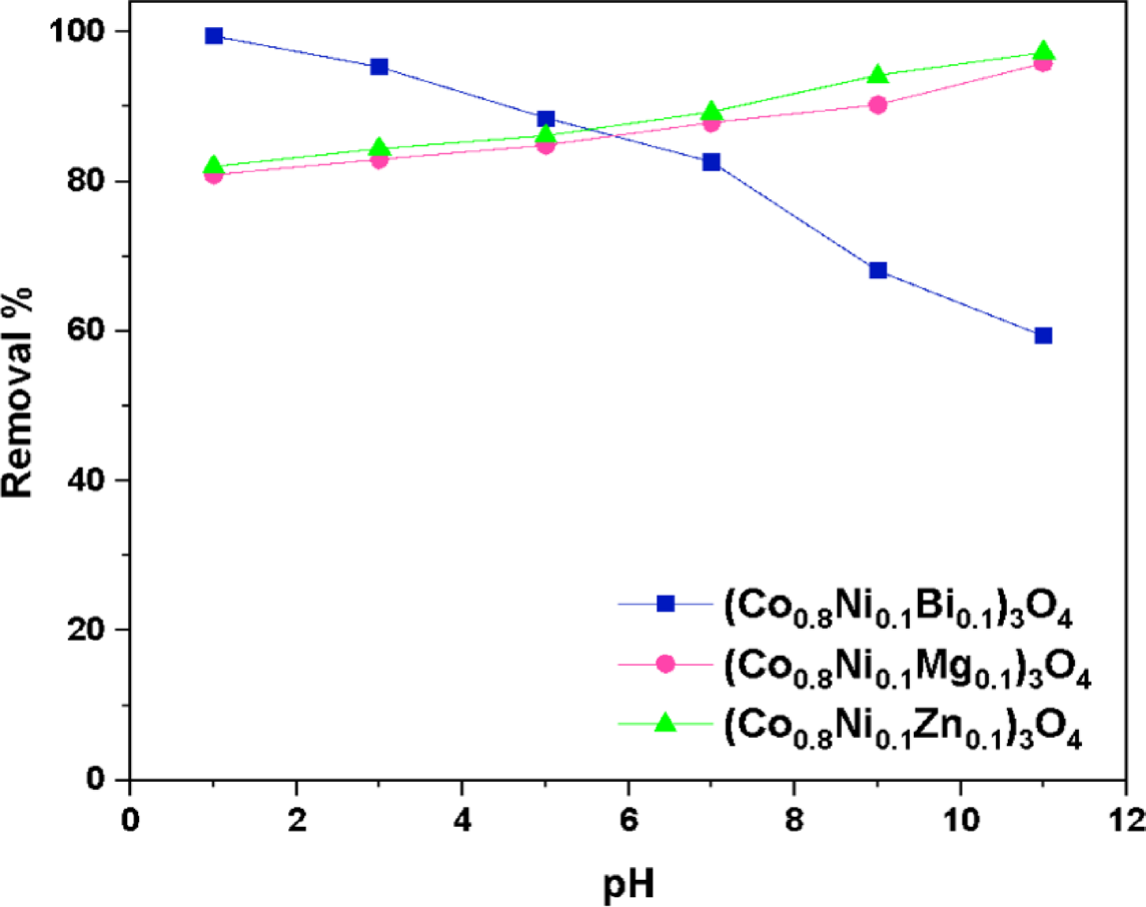
Effect of pH on the removal % of methylene blue in the presence of (Co_0.8_Ni_0.1_Bi_0.1_)_3_O_4_, (Co_0.8_Ni_0.1_Mg_0.1_)_3_O_4_, and (Co_0.8_Ni_0.1_Zn_0.1_)_3_O_4_ nanoparticles.

The influence of pH on the adsorption of methylene blue was examined by conducting the adsorption reaction in acidic, neutral, and basic media with pH values ranging from 1 to 11. The findings, illustrated in Fig. 19, indicate distinct behaviors between Zn, Mg, and Bi-doped (Co_0.8_Ni_0.1_ X _0.1_)_3_O_4_ nanoparticles. For (Co_0.8_Ni_0.1_Mg_0.1_)_3_O_4_ and (Co_0.8_Ni_0.1_Zn_0.1_)_3_O_4_ nanoparticles, the adsorption efficiency improves with increasing pH, reaching its highest removal percentage at pH 11, suggesting enhanced adsorption in an alkaline medium. Since methylene blue is a cationic dye, it is electrostatically attracted to negatively charged nanoparticles. Consequently, (Co_0.8_Ni_0.1_Mg_0.1_)_3_O_4_ and (Co_0.8_Ni_0.1_Zn_0.1_)_3_O_4_ nanoparticles are positively charged in acidic and neutral conditions but become negatively charged in alkaline conditions, explaining the improved adsorption in basic medium. It is worth noting that 95.8% and 97.2% of methylene blue dye are adsorbed onto (Co_0.8_Ni_0.1_Mg_0.1_)_3_O_4_ and (Co_0.8_Ni_0.1_Zn_0.1_)_3_O_4_ nanoparticles after a contact time of 30 min, respectively. In contrast, (Co_0.8_Ni_0.1_Bi_0.1_)_3_O_4_ nanoparticles exhibit superior adsorption performance in acidic conditions, achieving a maximum removal of 99.4% at pH 1. This might be due to the influence of Bi doping on the surface charge. Similar results were reported in a previous study by Dasuki et al.^76^. The study revealed that Bi doping caused the nanoparticles to become negatively charged, facilitating electrostatic attraction with the positively charged methylene blue dye in acidic media. This resulted in improved adsorption in acidic conditions for the Bi-doped samples. However, in basic media, the negative charge of the Bi-doped nanoparticles led to electrostatic repulsion, reducing adsorption efficiency.

#### Adsorption kinetics

**Fig. 20:**
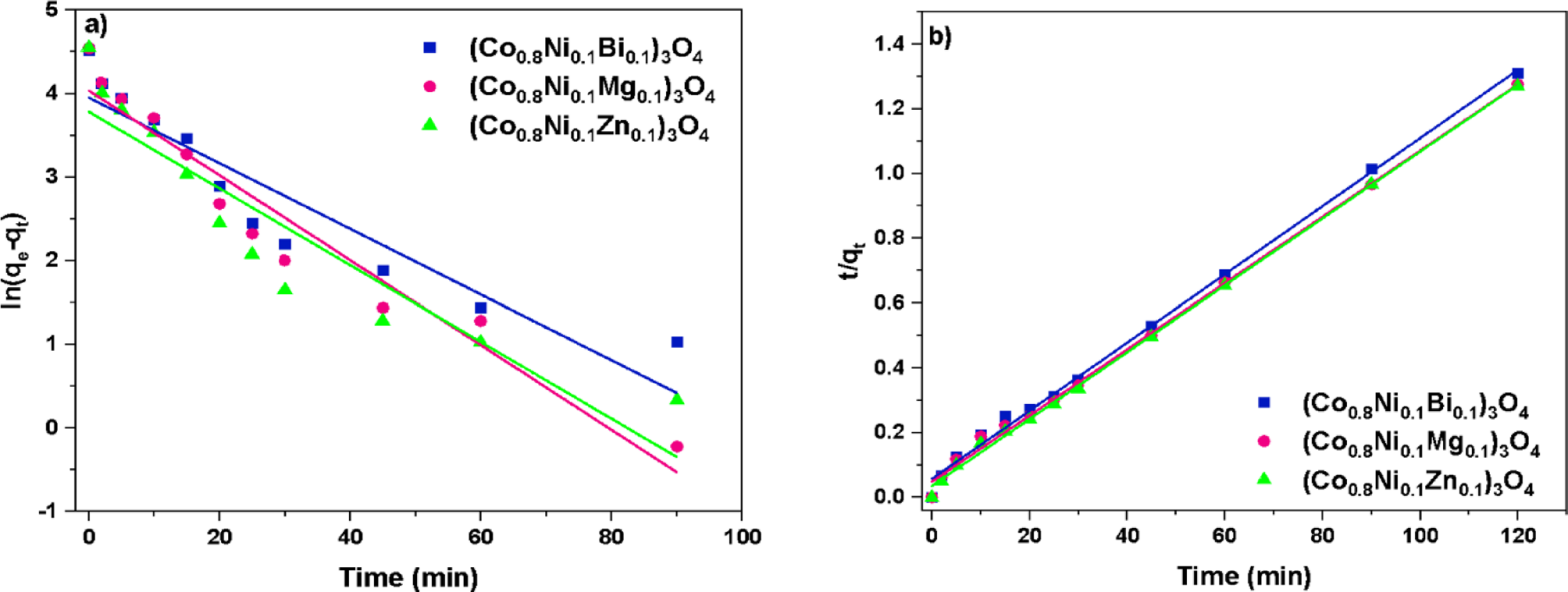
Fitting the experimental data with (a) first-order kinetics and (b) second-order kinetics models.

The experimental data are examined by both first and second-order kinetic models. The plots for the fitting of the kinetic models are illustrated in Fig. 20. The linearized expressions for these models are presented as follows^77^:

7 $${\text{ln }}\left( {q_{{\text{e}}} {-}q_{{\text{t}}} } \right) = - k_{{\text{1}}} t + {\text{ ln}}q_{{\text{e}}} ,\;{\text{and}}$$

8 $$\:\frac{t}{\:{q}_{\text{t}}}=\:\frac{t}{{q}_{\text{e}}}+\:\frac{1}{{k}_{2}{q}_{e}^{2}}$$

where *k*_1_ (min^−1^) and *k*_2_ (g.mg^−1^.min^−1^) signify the rate constant of the first-order and second-order models, respectively. The resultant values for the rate constants, adsorption capacity, and coefficient of determination (*R*^2^) are provided in Table 5. Notably, higher *R*^2^ values are evident for the second-order model in comparison to the first-order model. Consequently, the analysis of kinetic behavior suggests that the adsorption of methylene blue follows second-order kinetics. This finding aligns well with previous reports, where the adsorption of methylene blue onto Co_3_O_4_/SiO_2_ nanocomposites also followed second-order kinetics^78^. Additionally, the analysis reveals the highest rate constant (*k*_2_ = 0.0030 g.mg^−1^.min^−1^) for (Co_0.8_Ni_0.1_Zn_0.1_)_3_O_4_ nanoparticles, indicating that this composition demonstrates the highest adsorption efficiency for methylene blue removal among the prepared nanoparticles.

**Table 5:** Kinetic study parameters for the adsorption of methylene blue onto (Co_0.8_Ni_0.1_Bi_0.1_)_3_O_4_, (Co_0.8_Ni_0.1_Mg_0.1_)_3_O_4_, and (Co_0.8_Ni_0.1_Zn_0.1_)_3_O_4_ nanoparticles.

Nanoparticles		(Co_0.8_Ni_0.1_Bi_0.1_)_3_O_4_	(Co_0.8_Ni_0.1_Mg_0.1_)_3_O_4_	(Co_0.8_Ni_0.1_Zn_0.1_)_3_O_4_
First-order model	*k*_1_ (min^−1^)	0.0393	0.0507	0.0459
	*q*_e_ (mg.g^−1^)	52.04	56.63	43.93
	*R* ^2^	0.867	0.936	0.851
Second-order model	*k*_2_ (g.mg^−1^.min^−1^)	0.0019	0.0022	0.0030
	*q*_e_ (mg.g^−1^)	94.97	97.75	96.70
	*R* ^2^	0.996	0.997	0.998

Neither the first-order nor the second-order kinetic models adequately describe the diffusion mechanism involved in the adsorption process. Therefore, the intra-particle diffusion (IPD) model was applied to gain a deeper understanding of the adsorption behavior of methylene blue dye onto (Co_0.8_Ni_0.1_Bi_0.1_)_3_O_4_, (Co_0.8_Ni_0.1_Mg_0.1_)_3_O_4_, and (Co_0.8_Ni_0.1_Zn_0.1_)_3_O_4_ nanoparticles. The IPD model equation is expressed as follows^79^:

9 $$q_{{\text{t}}} = k_{{{\text{IPD}}}} {\text{t}}^{{{\text{1}}/{\text{2}}}} + {\text{ C}}.$$

**Fig. 21:**
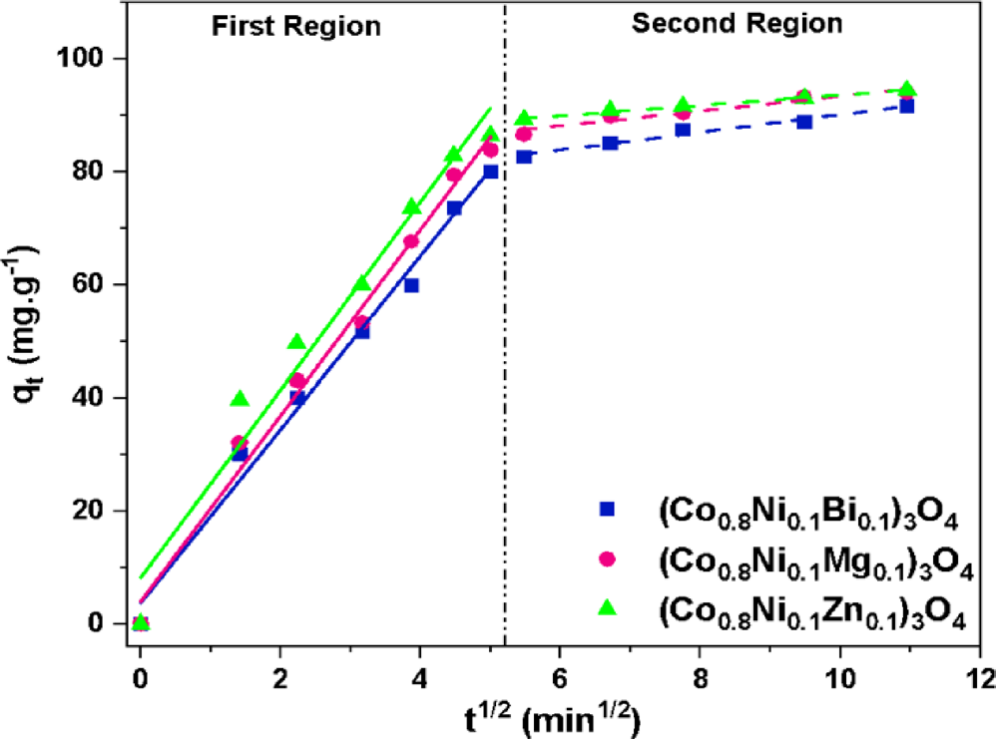
IPD plot for the adsorption of methylene blue onto Zn, Mg, and Bi-doped (Co_0.8_Ni_0.1_ X _0.1_)_3_O_4_ nanoparticles.

The plot of q^t^ versus t^1/2^, displayed in Fig. 21, permits the determination of the IPD rate constant (k_IPD_) and the thickness of the boundary layer (C) from the slope and intercept, respectively. The IPD model plot reveals two linear regions, indicating multiple stages in the adsorption process of methylene blue dye. As listed in Table 6, higher values of the IPD rate constant are observed in the first region compared to the second region. The initial rapid diffusion phase corresponds to the external surface adsorption of methylene blue, as evidenced by the first region of the IPD plot. In contrast, the subsequent slow adsorption phase is associated with the intraparticle diffusion of methylene blue within the pores of the (Co_0.8_Ni_0.1_Bi_0.1_)_3_O_4_, (Co_0.8_Ni_0.1_Mg_0.1_)_3_O_4_, and (Co_0.8_Ni_0.1_Zn_0.1_)_3_O_4_ nanoparticles, as revealed by the second region of the IPD plot^80^. The non-zero values of C, which reflect the boundary layer thickness, indicate the involvement of surface adsorption. This implies that intra-particle diffusion is not the only rate-limiting step in the process.

**Table 6:** Parameters extracted from the IPD model for the adsorption of methylene blue onto Zn, Mg, and Bi-doped (Co_0.8_Ni_0.1_X _0.1_)_3_O_4_ nanoparticles.

Doping element		Bi	Mg	Zn
First Region	*k*_IPD_ (mg.g^−1^.min^−1/2^)	15.36	16.45	16.63
	*C* (mg.g^−1^)	3.63	3.92	8.11
	*R* ^2^	0.99	0.99	0.96
Second Region	*k*_IPD_ (mg.g^−1^.min^−1/2^)	1.57	1.32	0.91
	*C* (mg.g^−1^)	74.41	80.18	84.43
	*R* ^2^	0.97	0.93	0.99

#### Adsorption isotherm

**Fig. 22:**
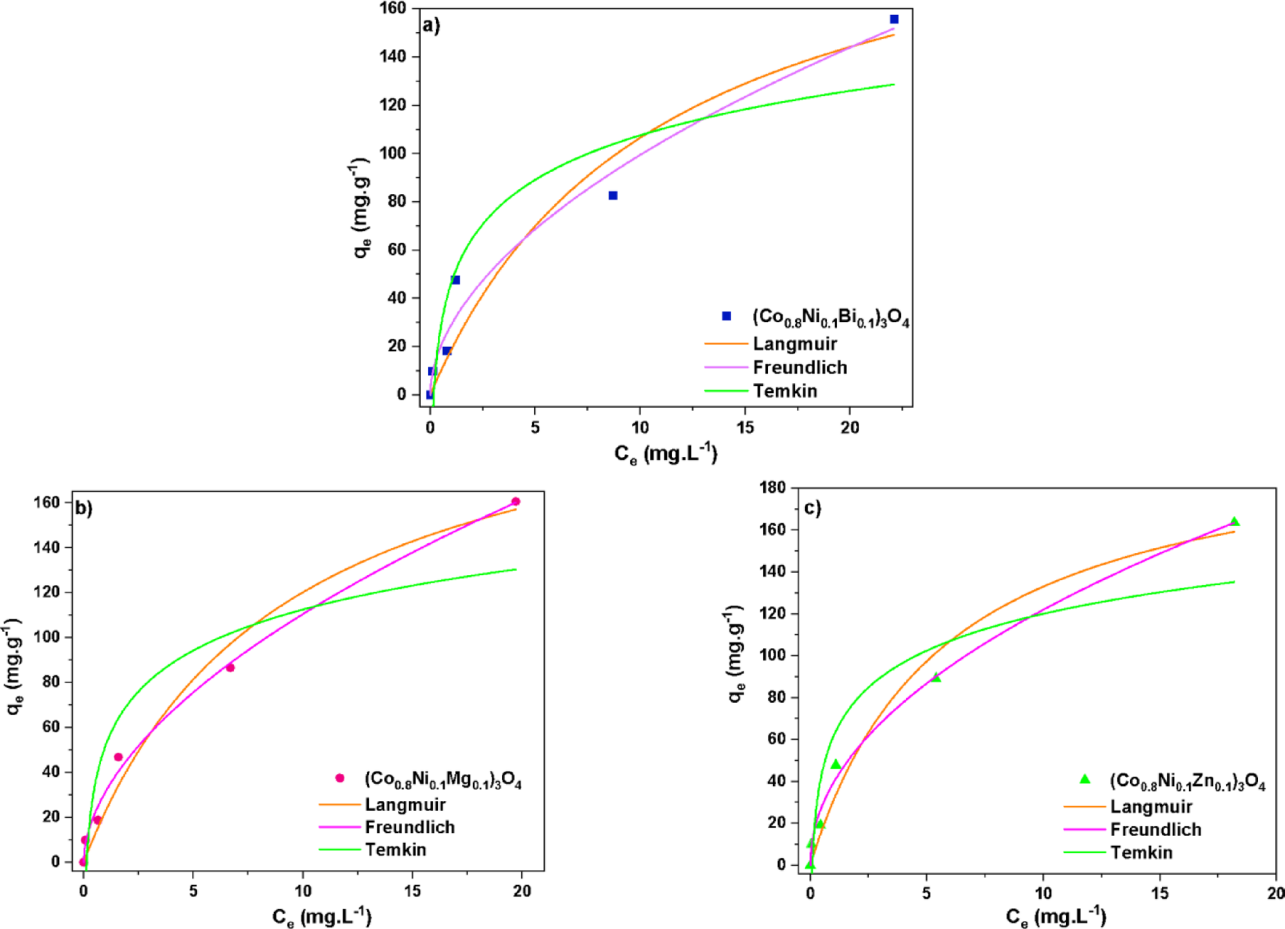
Non-linear adsorption isotherm fitting plots for the adsorption of methylene blue onto (a) Bi, (b) Mg, and (c) Zn-doped (Co_0.8_Ni_0.1_ X _0.1_)_3_O_4_ nanoparticles.

To comprehend the adsorption mechanism and analyze the relationship between the dye and the prepared nanoparticles, it is critical to examine the adsorption isotherms. Consequently, non-linear Langmuir, Freundlich, and Temkin isotherms were employed. Figure 22 demonstrates the fitted isotherm plots, whereas Table 7 summarizes the corresponding parameters obtained from the models. The Langmuir isotherm model implies that there are no intermolecular interactions between adsorbate molecules, suggesting that methylene blue dye adsorption occurs as a monolayer on the surface of the adsorbent. The Langmuir isotherm equation is represented as follows^67^:

10 $$\:{\:q}_{\text{e}}=\:\frac{{Q}_{\text{L}}{b}_{\text{L}}{C}_{\text{e}}}{1+\:{b}_{\text{L}}{C}_{\text{e}}},$$

where *Q*_L_ and *b*_L_ denote the Langmuir constant and the maximum monolayer adsorption capacity, respectively. In contrast, the Freundlich isotherm model suggests the formation of multiple adsorption layers and is applicable to surfaces with heterogeneous adsorption characteristics. It is particularly relevant when the adsorption surface is non-uniform or contains sites with varying affinities for the adsorbate molecules^81^. The Freundlich isotherm equation is expressed as follows^67^:

11 $$\:{\:\:\:\:\:\:\:\:\:\:\:\:\:\:\:\:\:\:\:\:\:\:\:\:\:\:\:\:\:\:\:\:\:\:\:\:\:\:\:\:\:\:\:\:\:\:\:\:\:\:\:\:\:\:\:\:\:\:\:\:\:\:\:\:q}_{\text{e}}=\:{\:K}_{\text{F}}\times\:\left({C}_{e}^{\frac{1}{n}}\right).$$

The parameters n and *K*_F_ symbolize the adsorption intensity and the Freundlich constant, respectively. The Temkin model takes into account the interactions between adsorbate and adsorbent molecules and is described by the following equation^77^:

12 $$\:{\:q}_{\text{e}}=\:\frac{RT}{{b}_{T}}ln\:\left({K}_{\text{T}}{C}_{\text{e}}\right)\:,$$

the adsorption heat coefficient is signified by *b*_T_, whereas the equilibrium binding constant is denoted as *K*_T_. Among the models analyzed, the Freundlich isotherm model showed the highest R² values.

It is clear that (*R*^2^) values for the second-order kinetics are around 0.99 for all investigated samples in comparison with those values determined from first-order kinetics; (*R*^2^) varied between 0.85 and 0.94. Najdanović et al.^82^, reported that when (*R*^2^) was higher than 0.99 for both first and pseudo-second order models, they used a new parameter, mean relative deviation (MRD), to show better agreement with the experimental results. This indicates that the Freundlich isotherm model provides the best fit for describing the adsorption process of methylene blue onto the synthesized nanoparticles. In addition, (Co_0.8_Ni_0.1_Zn_0.1_)_3_O_4_ nanoparticles demonstrated the highest values for the Freundlich constant and adsorption intensity. Improved adsorption is associated with n values falling within the range of 1–10^83^. Therefore, the favorable adsorption of methylene blue dye onto the synthesized adsorbents is indicated by the recorded n values, which fall within this range.

**Table 7:** Parameters derived from the fitting of non-linear Langmuir, Freundlich, and Temkin adsorption isotherms for (Co_0.8_Ni_0.1_ X _0.1_)_3_O_4_ nanoparticles.

Doping element (X)		Bi	Mg	Zn
Experimental *q*_e_ **(mg.g**^**−1**^**)**		91.6	94	94.4
Langmuir	*Q*_L_ (mg.g^−1^)	223.19	229.79	209.71
	*b*_L_ **(L.mg**^**−1**^**)**	0.091	0.11	0.17
	*R* ^2^	0.92	0.97	0.97
Freundlich	*K*_F_ ((mg.g^−1^)(mg.L^−1^)^1/n^)	29.06	31.36	39.47
	n	1.87	1.83	2.04
	*R* ^2^	0.97	0.99	0.99
Temkin	*K*_T_ (L.mg^−1^)	5.71	7.16	11.35
	*b*_T_ **(**J.mol^−1^**)**	93.09	94.01	97.60
	*R* ^2^	0.85	0.83	0.85

## Conclusions

Single spinel cubic (Co_0.8_Ni_0.1_X _0.1_)_3_O_4_ nanoparticles (X = Zn, Mg, and Bi) were synthesized using the chemical co-precipitation technique, with a crystallite size range between 17 and 23 nm. The morphological analysis, SEM, and TEM images illustrated the formation of agglomerated particles that may be formed by the high surface energy of the small-dimension particles. The elemental composition analysis, EDX, and XPS analysis confirmed the presence of all the main core elements for Zn- and Mg-doped samples. However, a secondary phase (BiOCl phase) was detected in the FTIR spectra of the Bi-doped sample, with an additional Cl element seen in the EDX and XPS spectra. Furthermore, the high O_V_/O_L_ belongs to the higher surface oxygen vacancy sites, present at a higher concentration in the Zn-doped sample than in the Mg and Bi-doped samples. The charge transfers between cobalt and oxygen were detected by the two adsorption bands per sample in the UV-vis analysis (the first band ≈ 280 nm relates to Co^2+^ and O^2−^, while the second band ≈ 500 nm associated with Co^3+^ and O^2−^), and through the blue and green emission peaks in the PL analysis. Five main stretching peaks were detected in the Raman spectroscopy, confirming the spinel structure of Co_3_O_4_ nanocrystals for all samples. However, the Bi-doped Raman peaks were red-shifted and broadened, which refers to the induced lattice contraction and distortion due to the formation of the BiOCl secondary phase. The adsorption activity of the synthesized NPs toward methylene blue was comprehensively evaluated. The optimal contact time was found to be 30 min, after which equilibrium was reached, with (Co_0.8_Ni_0.1_Zn_0.1_)_3_O_4_ displaying the highest adsorption capacity (94.4 mg·g⁻¹). Kinetic studies revealed that the adsorption process follows a pseudo-second-order model, with the highest rate constant observed for the Zn-doped sample. The intra-particle diffusion model further demonstrated that the adsorption proceeds in two stages: a rapid surface adsorption followed by slower diffusion within the pores. Increasing the initial dye concentration up to 100 mg. L^−1^ led to decreased removal efficiency but enhanced adsorption capacity, reaching up to 163.6 mg·g⁻¹ for the Zn-doped sample. Isotherm modeling confirmed that the adsorption behavior fits best with the Freundlich isotherm, indicating a multilayer adsorption process on a heterogeneous surface. The effect of pH showed that adsorption efficiency increases in basic media for Zn and Mg-doped samples, reaching a maximum at pH 11. In contrast, the Bi-doped sample demonstrated optimal performance in an acidic medium, with a decrease in adsorption observed in both highly acidic and highly basic conditions. This result is attributed to the influence of Bi doping on altering the surface charge. For future studies, the adsorption performance of the synthesized nanoparticles could be evaluated using real water samples with organic pollutants to enhance the direct applicability of the study.

## Data Availability

The data generated or analyzed during this study are available upon reasonable request from the corresponding author.

## References

[1] Lima, A. F. Interpretation of the optical absorption spectrum of Co3O4 with normal spinel structure from first principles calculations. *J. Phys. Chem. Solids*. **75**, 148–152 (2014).

[2] Gangopadhyay, S., Hadjipanayis, G. C., Sorensen, C. M. & Klabunde, K. J. Exchange anisotropy in oxide passivated Co fine particles. *J. Appl. Phys.* **73**, 6964–6966 (1993).

[3] He, L., Chen, C., Wang, N., Zhou, W. & Guo, L. Finite size effect on néel temperature with Co3O4 nanoparticles. *J. Appl. Phys.* **102** (2007).

[4] Resnick, D. A. et al. Magnetic properties of Co3O4 nanoparticles mineralized in *Listeria innocua* Dps. *J. Appl. Phys.* **99** (2006).

[5] Salabaş, E. L., Rumplecker, A., Kleitz, F., Radu, F. & Schüth, F. Exchange anisotropy in nanocasted Co3 O4 nanowires. *Nano Lett.* **6**, 2977–2981 (2006). 17163743 10.1021/nl060528n

[6] Hassaan, M. A., Elkatory, M. R., El-Nemr, M. A. & Ragab, S. El nemr, A. Optimization strategy of Co3O4 nanoparticles in biomethane production from seaweeds and its potential role in direct electron transfer and reactive oxygen species formation. *Sci. Rep.* **14**, 5075 (2024). 38429365 10.1038/s41598-024-55563-y PMC11319461

[7] Lavanya, S. et al. Enhanced structural, optical, and photo sensing properties of Ni-doped Co3O4 thin films prepared by nebulizer spray pyrolysis method. *Phys. B: Condens. Matter*. **649**, 414492 (2023).

[8] Ren, Z. et al. Low temperature propane oxidation over Co3O4 based nano-array catalysts: Ni Dopant effect, reaction mechanism and structural stability. *Appl. Catal. B*. **180**, 150–160 (2016).

[9] Barbouch, Z. et al. Unravelling the effects of Zn doping on the physical properties of Co3O4 thin films: experimental study and numerical simulation based on the AZO/ZnS/Co3O4:Zn/Mo/SLG solar cells. *Opt. Quant. Electron.* **56**, 1441 (2024).

[10] Sundararajan, M. et al. Rapid synthesis and magnetic property characterization of Mg2+ doped Co3O4 nanostructures. *Inorg. Nano-Metal Chem.* **52**, 996–1002 (2022).

[11] Deeloed, W. et al. A systematic variation in cationic distribution and its influence on the magnetization of mixed-metal (nickel and zinc) Cobaltite spinels. *Mater. Res. Express*. **7**, 096104 (2020).

[12] Trivedi, Y. et al. Biochar potential for pollutant removal during wastewater treatment: A comprehensive review of separation mechanisms, technological integration, and process analysis. *Desalination* **600**, 118509 (2025).

[13] Taher, A. et al. Applications of nano composites for heavy metal removal from water by adsorption: Mini review. *J. Nanostruct.* **14**, 1239–1251 (2024).

[14] Tariq, A. & Mushtaq, A. Untreated wastewater reasons and causes: A review of most affected areas and cities. *Int. J. Chem. Biochem. Sci.* **23**, 121–143 (2023).

[15] Iqbal, S. et al. Magnetically recyclable Mn and Co doped r-GO based LaFeO3 nano-composite for tuning the optical and solar light driven photo-catalytic applications. *J. Water Process. Eng.* **70**, 107110 (2025).

[16] Iqbal, S. et al. Gd and Co-substituted LaNiO3 and their nanocomposites with r-GO for photocatalytic applications. *Diam. Relat. Mater.* **110**, 108119 (2020).

[17] Rashid, R., Shafiq, I., Akhter, P., Iqbal, M. J. & Hussain, M. A state-of-the-art review on wastewater treatment techniques: the effectiveness of adsorption method. *Environ. Sci. Pollut Res.* **28**, 9050–9066 (2021). 10.1007/s11356-021-12395-x 33483933

[18] Majeed, H. J. et al. Synthesis and application of novel sodium carboxy Methyl cellulose-g-poly acrylic acid carbon Dots hydrogel nanocomposite (NaCMC-g-PAAc/ CDs) for adsorptive removal of malachite green dye. *Desalination Water Treat.* **320**, 100822 (2024).

[19] Tsyats’ko, V. V., Gokov, S. P. & Kazarinov, Y. G. *Interaction of Fluxes of Fast and Thermal Neutrons with an Aqueous Solution of Organic Dye Methylene Blue Containing and not Containing Boric Acid.* Problems of Atomic Science and Technology (2023).

[20] Oladoye, P. O., Ajiboye, T. O., Omotola, E. O. & Oyewola, O. J. Methylene blue dye: toxicity and potential elimination technology from wastewater. *Results Eng.* **16**, 100678 (2022).

[21] Katheresan, V., Kansedo, J. & Lau, S. Y. Efficiency of various recent wastewater dye removal methods: A review. *J. Environ. Chem. Eng.* **6**, 4676–4697 (2018).

[22] Al Nafiey, A. et al. Reduced graphene oxide decorated with Co3O4 nanoparticles (rGO-Co3O4) nanocomposite: a reusable catalyst for highly efficient reduction of 4-nitrophenol, and cr (VI) and dye removal from aqueous solutions. *Chem. Eng. J.* **322**, 375–384 (2017).

[23] Uddin, M. K. & Baig, U. Synthesis of Co3O4 nanoparticles and their performance towards Methyl orange dye removal: characterisation, adsorption and response surface methodology. *J. Clean. Prod.* **211**, 1141–1153 (2019).

[24] Nagarajan, V. & Chandiramouli, R. A DFT study on adsorption behaviour of CO on Co3O4 nanostructures. *Appl. Surf. Sci.* **385**, 113–121 (2016).

[25] Zeid, E. F. A., Obiedallah, F. M., Abu-Sehly, A. H. & Mohamed, W. A. Abd El-Aal, M. A comparative study of single and bi-doped Co3O4 nanocatalysts for the photodegradation of Methyl orange dye. *J. Mol. Struct.* **1293**, 136203 (2023).

[26] Kathiravan, P., Thillaivelavan, K., Viruthagiri, G. & Shanmugha Sundaram, P. Effect of Cobalt addition on structural, optical, morphological and magnetic properties of NiO nanoparticles prepared by chemical precipitation method. *Inorg. Chem. Commun.* **161**, 112057 (2024).

[27] Rmeid, S. et al. Removal of radioactive Co(II) and Sr(II) using (Co0.5Zn0.5)Fe2O4 nanoparticles co-doped with barium and antimony. *Ceram. Int. S*10.1016/j.ceramint.2024.11.433 (2024).

[28] Lutterotti, L. M. A. U. D. - Materials Analysis Using Diffraction (and more). https://luttero.github.io/maud/ (2023).

[29] Gao, Y. et al. BiOCl for efficient visible light photocatalytic CO2 reduction with abundant oxygen vacancies and WO3 as Pn heterojunctions. *J. Alloys Compd.* **983**, 173824 (2024).

[30] Wosylus, A., Hoffmann, S., Schmidt, M. & Ruck, M. In-situ study of the solid-gas reaction of BiCl3 to BiOCl via the intermediate hydrate BiCl3 ·H2 O. *Eur. J. Inorg. Chem.* **2010**, 1469–1471 (2010).

[31] Li, H., Liu, J., Qian, J., Li, Q. & Yang, J. Preparation of Bi-doped TiO2 nanoparticles and their visible light photocatalytic performance. *Chin. J. Catal.* **35**, 1578–1589 (2014).

[32] Aadil, M., Zulfiqar, S., Warsi, M. F., Agboola, P. O. & Shakir, I. Free-standing urchin-like nanoarchitectures of Co3O4 for advanced energy storage applications. *J. Mater. Res. Technol.* **9**, 12697–12706 (2020).

[33] Shannon, R. D. Revised effective ionic radii and systematic studies of interatomic distances in halides and chalcogenides. *Found. Crystallogr.* **32**, 751–767 (1976).

[34] Yu, Y. et al. Facile synthesis of Ni3+/Co3+ Ion-Doped Zn2SnO4 microspheres toward efficient photocatalytic CO2 reduction. *Appl. Sci.* **13**, 13193 (2023).

[35] Aridi, A. et al. Influence of Gd3+ ion doping on structural, optical, and magnetic properties of (Mg–Ni–Co) nanoferrites. *J. Nanopart. Res.* **25**, 228 (2023).

[36] Riazian, M. Photocatalytic activity and nano structural investigation on Co3O4 nanoparticles. *Nanochemistry Res.* **5**, 46–58 (2020).

[37] Sarfraz, A. & Hasanain, S. Size dependence of magnetic and optical properties of Co3O4 nanoparticles. *Acta Phys. Pol., A*. **125**, 139–144 (2014).

[38] Abdallah, A. M. & Awad, R. Sm and Er partial alternatives of Co in Co3O4 nanoparticles: probing the physical properties. *Phys. B: Condens. Matter*. **608**, 412898 (2021).

[39] Wang, Z., Liu, Q., Yu, J., Wu, T. & Wang, G. Surface structure and catalytic behavior of silica-supported copper catalysts prepared by impregnation and sol–gel methods. *Appl. Catal. A*. **239**, 87–94 (2003).

[40] Stella, C., Soundararajan, N. & Ramachandran, K. Structural, optical, and magnetic properties of Mn and Fe-doped Co3O4 nanoparticles. *AIP Adv.* **5** (2015).

[41] Lang, D., Cheng, F. & Xiang, Q. Enhancement of photocatalytic H 2 production activity of cds nanorods by cobalt-based Cocatalyst modification. *Catal. Sci. Technol.* **6**, 6207–6216 (2016).

[42] Huang, Q. et al. Direct fabrication of lamellar self-supporting Co3O4/N/C peroxymonosulfate activation catalysts for effective aniline degradation. *Chem. Eng. J.* **313**, 1088–1098 (2017).

[43] Das, A. et al. Local structure distortion in mn, Zn doped cu₂v₂o₇: supercapacitor performance and emergent Spin-Phonon coupling. *Adv. Mater.* **37**, 2416644 (2025). 10.1002/adma.202416644 39865953

[44] Mori, H. & Yoshida, H. Effect of hot isostatic pressing treatment on the thermoelectric power factors of zinc oxides. *Sci. Rep.* **14**, 31367 (2024). 39733110 10.1038/s41598-024-82880-z PMC11682204

[45] Mittal, V. K. et al. Solid state synthesis of Mg–Ni ferrite and characterization by XRD and XPS. *J. Nucl. Mater.* **335**, 302–310 (2004).

[46] Ginting, R. T. et al. A simple approach Low-Temperature solution process for Preparation of Bismuth-Doped ZnO nanorods and its application in hybrid solar cells. *J. Phys. Chem. C*. **120**, 771–780 (2016).

[47] Shrestha, S., Wang, B. & Dutta, P. Nanoparticle processing: Understanding and controlling aggregation. *Adv. Colloid Interface Sci.* **279**, 102162 (2020). 32334131 10.1016/j.cis.2020.102162

[48] WS, R. Imagej, us national institutes of health, Bethesda, Maryland, USA. http://imagej.nih.gov/ij/ (2011).

[49] Al-Senani, G. M. & Al-Saeedi, S. I. The use of synthesized CoO/Co3O4 nanoparticles as a corrosion inhibitor of low-carbon steel in 1 M HCl. *Materials* **15**, 3129 (2022). 35591463 10.3390/ma15093129 PMC9104794

[50] Labban, W., Aridi, A., Habanjar, K. & Awad, R. Exploring the influence of heat treatment duration and temperature on the properties and adsorption performance of Sn0.5Ni0.3Mn0.2Fe2O4 nanoparticles. *Environ. Sci. Pollut Res.* **32**, 11654–11673 (2025). 10.1007/s11356-025-36397-1 40234317

[51] Qin, Y., Guo, L., Liu, C., Zhang, J. & Wang, Q. Tailoring coordinated steps with Ni-Substituted Co3o4 asymmetric active unit for durable and efficient acidic water oxidation. SSRN Scholarly Paper. 10.2139/ssrn.5141976 (2025). 10.1016/j.jcis.2025.137828 40359633

[52] Tang, W. et al. Boosting catalytic propane oxidation over PGM-free Co3O4 nanocrystal aggregates through chemical leaching: A comparative study with Pt and Pd based catalysts. *Appl. Catal. B*. **226**, 585–595 (2018).

[53] Adesuji, E. et al. Pt-Co3O4 superstructures by One-Pot reduction/precipitation in bicontinuous microemulsion for electrocatalytic oxygen evolution reaction. *Catalysts* **10**, 1311 (2020).

[54] Ramana, C. V., Massot, M. & Julien, C. M. XPS and Raman spectroscopic characterization of LiMn2 O4 spinels. *Surf. Interface Anal.* **37**, 412–416 (2005).

[55] Hudy, C. et al. Catalytic performance of mixed M x Co 3–x O 4 (M = Cr, fe, mn, ni, cu, Zn) spinels obtained by combustion synthesis for Preferential carbon monoxide oxidation (CO-PROX): insights into the factors controlling catalyst selectivity and activity. 10.1039/D2CY00388K (2022).

[56] Stanojević, Z. M., Romčević, N. & Stojanović, B. Spectroscopic study of spinel ZnCr2O4 obtained from mechanically activated ZnO–Cr2O3 mixtures. *J. Eur. Ceram. Soc.* **27**, 903–907 (2007).

[57] Wang, Y., Wei, X., Hu, X., Zhou, W. & Zhao, Y. Effect of formic acid treatment on the structure and catalytic activity of Co3O4 for N2O decomposition. *Catal. Lett.* **149**, 1026–1036 (2019).

[58] Farhadi, S., Safabakhsh, J. & Zaringhadam, P. Synthesis, characterization, and investigation of optical and magnetic properties of Cobalt oxide (Co3O4) nanoparticles. *J. Nanostruct. Chem.* **3**, 69 (2013).

[59] Kumar, K. V. Tunable optical bandgap of gadolinium substituted nickel-zinc ferrite nanoparticles-effect of calcination temperature on its optical parameters. *Adv. Mater. Phys. Chem.* **12**, 33–45 (2022).

[60] Siddique, M. N., Ali, T., Ahmed, A. & Tripathi, P. Enhanced electrical and thermal properties of pure and Ni substituted ZnO nanoparticles. *Nano-Structures Nano-Objects*. **16**, 156–166 (2018).

[61] Hu, X. et al. Synthesis of novel ternary heterogeneous BiOCl/TiO2/sepiolite composite with enhanced visible-light-induced photocatalytic activity towards Tetracycline. *J. Colloid Interface Sci.* **533**, 238–250 (2019). 30165301 10.1016/j.jcis.2018.08.077

[62] Lakehal, A., Benrabah, B., Bouaza, A., Dalache, C. & Hadj, B. Tuning of the physical properties by various transition metal doping in Co3O4: TM (TM= ni, mn, Cu) thin films: A comparative study. *Chin. J. Phys.* **56**, 1845–1852 (2018).

[63] Muradov, M. B. et al. Synthesis of CuxCo3–xO4 nanoparticles by a sonochemical method and characterization of structural and optical properties and photocatalytic activity for the degradation of methylene blue. *RSC Adv.* **14**, 1082–1093 (2024). 38174276 10.1039/d2ra08060e PMC10759309

[64] Ghobashy, M. M., Sharshir, A. I., Zaghlool, R. A. & Mohamed, F. Investigating the impact of electron beam irradiation on electrical, magnetic, and optical properties of XLPE/Co3O4 nanocomposites. *Sci. Rep.* **14**, 4829 (2024). 38413685 10.1038/s41598-024-55085-7 PMC10899620

[65] Raja, V., Puvaneswaran, S. K. & Swaminathan, K. Unique and hierarchically structured novel Co3O4/NiO nanosponges with superior photocatalytic activity against organic contaminants. *Front. Mater. Sci.* **11**, 375–384 (2017).

[66] Kostić, M. et al. Ultrasound-assisted synthesis of a new material based on MgCoAl-LDH: characterization and optimization of sorption for progressive treatment of water. *Environ. Technol. Innov.* **26**, 102358 (2022).

[67] Kassem, S., AlHajjar, N., Aridi, A. & Awad, R. Identification of structural and optical properties and adsorption performance of (Cd0.4Ni0.4Mn0.2)Fe2-xRuxO4 nanoparticles for the removal of congo red dye. *Arab. J. Chem.* **17**, 105477 (2024).

[68] El-Dossoki, F., Rady, S. & Hosny, N. Synthesis of Co3O4 nanoparticles from amino acids mixed ligands and its adsorption properties. *Alfarama J. Basic. Appl. Sci.* **1**, 1–12 (2020).

[69] Xu, Z., Zhang, C., Zhang, Y., Gu, Y. & An, Y. BiOCl-based photocatalysts: synthesis methods, structure, property, application, and perspective. *Inorg. Chem. Commun.* **138**, 109277 (2022).

[70] Essa, W. K. Methylene blue removal by copper oxide nanoparticles obtained from green synthesis of Melia azedarach: kinetic and isotherm studies. *Chemistry* **6**, 249–263 (2024).

[71] Ahmadi, S. Removal of methylene blue on zinc oxide nanoparticles: nonlinear and linear adsorption isotherms and kinetics study. *Sigma J. Eng. Nat. Sci.* **38**, 289–303 (2020).

[72] Kavaz, D. Effective methylene dye removal from contaminated water using zinc oxide nanoparticles fabricated from the green synthesis of Laurus nobilis leaf. *J. Chem. Soc. Pak.* **46** (2024).

[73] Jaramillo-Fierro, X. & Cuenca, G. Enhancing methylene blue removal through adsorption and photocatalysis—a study on the Go/ZnTiO3/TiO2 composite. *Int. J. Mol. Sci.* **25**, 4367 (2024). 38673952 10.3390/ijms25084367 PMC11049837

[74] Velinov, N. et al. The influence of various solvents’ Polarity in the synthesis of wood Biowaste sorbent: evaluation of dye sorption. *Biomass Conv Bioref*. **13**, 8139–8150 (2023).

[75] Adewuyi, A., Gervasi, C. A. & Mirífico, M. V. Synthesis of strontium ferrite and its role in the removal of Methyl orange, phenolphthalein and bromothymol blue from laboratory wastewater. *Surf. Interfaces*. **27**, 101567 (2021).

[76] Dasuki, D. et al. Adsorptive performance of bismuth-doped Ni-Zn-Co ferrite nanoparticles for the removal of methylene blue dye. *Environ. Sci. Pollut Res.* **32**, 23–42 (2024). 10.1007/s11356-024-35734-0 39663304

[77] Aridi, A. et al. Enhanced adsorption performance of magnetic Ni0.5Zn0.5Fe2O4/Zn0.95Co0.05O nanocomposites for the removal of malachite green dye. *Environ. Sci. Pollut Res.* **30**, 58399–58411 (2023). 10.1007/s11356-023-26608-y 36991201

[78] Abdel Ghafar, H. H., Ali, G. A. M., Fouad, O. A. & Makhlouf, S. A. Enhancement of adsorption efficiency of methylene blue on Co 3 O 4 /SiO 2 nanocomposite. *Desalination Water Treat.* **53**, 2980–2989 (2015).

[79] Li, Y. et al. Adsorption and electrochemical behavior investigation of Methyl blue onto magnetic nickel-magnesium ferrites prepared via the rapid combustion process. *J. Alloys Compd.* **885**, 160969 (2021).

[80] Kostić, M. M. et al. Effects of ultrasound on removal of ranitidine hydrochloride from water by activated carbon based on Lagenaria siceraria. *Environ. Eng. Sci.* **36**, 237–248 (2019).

[81] Mashkoor, F., Nasar, A. & Magsorbents Potential candidates in wastewater treatment technology – A review on the removal of methylene blue dye. *J. Magn. Magn. Mater.* **500**, 166408 (2020).

[82] Najdanović, S. M. et al. Effect of electrochemical synthesis parameters on the morphology, crystal and chemical structure, and sorption efficiency of basic bismuth nitrates. *Molecules* **30** (2025). 10.3390/molecules30051020 PMC11901594 40076245

[83] Zhang, P. et al. High efficiency removal of methylene blue using SDS surface-modified ZnFe2O4 nanoparticles. *J. Colloid Interface Sci.* **508**, 39–48 (2017). 28818655 10.1016/j.jcis.2017.08.025

